# Genome-Wide Association Studies in an Isolated Founder Population from the Pacific Island of Kosrae

**DOI:** 10.1371/journal.pgen.1000365

**Published:** 2009-02-06

**Authors:** Jennifer K. Lowe, Julian B. Maller, Itsik Pe'er, Benjamin M. Neale, Jacqueline Salit, Eimear E. Kenny, Jessica L. Shea, Ralph Burkhardt, J. Gustav Smith, Weizhen Ji, Martha Noel, Jia Nee Foo, Maude L. Blundell, Vita Skilling, Laura Garcia, Marcia L. Sullivan, Heather E. Lee, Anna Labek, Hope Ferdowsian, Steven B. Auerbach, Richard P. Lifton, Christopher Newton-Cheh, Jan L. Breslow, Markus Stoffel, Mark J. Daly, David M. Altshuler, Jeffrey M. Friedman

**Affiliations:** 1 The Rockefeller University, New York, New York, United States of America; 2 Department of Molecular Biology, Massachusetts General Hospital, Boston, Massachusetts, United States of America; 3 Program in Medical and Population Genetics, The Broad Institute of Harvard and MIT, Cambridge, Massachusetts, United States of America; 4 Center for Human Genetics Research, Massachusetts General Hospital, Boston, Massachusetts, United States of America; 5 Social, Genetic, and Developmental Psychiatry Centre, Institute of Psychiatry, King's College London, United Kingdom; 6 Department of Genetics, Yale University School of Medicine, New Haven, Connecticut, United States of America; 7 Kosrae Department of Health Services, Kosrae, Federated States of Micronesia; 8 Department of Health and Human Services, Heath Resources and Services Administration, New York, New York, United States of America; 9 Howard Hughes Medical Institute, Chevy Chase, Maryland, United States of America; 10 Cardiovascular Research Center, Massachusetts General Hospital, Boston, Massachusetts, United States of America; 11 Department of Medicine, Harvard Medical School, Boston, Massachusetts, United States of America; The University of Queensland, Australia

## Abstract

It has been argued that the limited genetic diversity and reduced allelic heterogeneity observed in isolated founder populations facilitates discovery of loci contributing to both Mendelian and complex disease. A strong founder effect, severe isolation, and substantial inbreeding have dramatically reduced genetic diversity in natives from the island of Kosrae, Federated States of Micronesia, who exhibit a high prevalence of obesity and other metabolic disorders. We hypothesized that genetic drift and possibly natural selection on Kosrae might have increased the frequency of previously rare genetic variants with relatively large effects, making these alleles readily detectable in genome-wide association analysis. However, mapping in large, inbred cohorts introduces analytic challenges, as extensive relatedness between subjects violates the assumptions of independence upon which traditional association test statistics are based. We performed genome-wide association analysis for 15 quantitative traits in 2,906 members of the Kosrae population, using novel approaches to manage the extreme relatedness in the sample. As positive controls, we observe association to known loci for plasma cholesterol, triglycerides, and C-reactive protein and to a compelling candidate loci for thyroid stimulating hormone and fasting plasma glucose. We show that our study is well powered to detect common alleles explaining ≥5% phenotypic variance. However, no such large effects were observed with genome-wide significance, arguing that even in such a severely inbred population, common alleles typically have modest effects. Finally, we show that a majority of common variants discovered in Caucasians have indistinguishable effect sizes on Kosrae, despite the major differences in population genetics and environment.

## Author Summary

Isolated populations have contributed to the discovery of loci with simple Mendelian segregation and large effects on disease risk or trait variation. We hypothesized that the use of isolated populations might also facilitate the discovery of common alleles contributing to complex traits with relatively larger effects. However, the use of association analyses to map common loci influencing trait variation in large, inbred cohorts introduces analytic challenges, as extensive relatedness between subjects violates the assumptions of independence upon which traditional association test statistics are based. We developed an analytic strategy to perform genome-wide association studies in an inbred family containing over 2,800 individuals from the island of Kosrae, Federated States of Micronesia. No alleles with large effect were observed with strong statistical support in any of the 15 traits examined, suggesting that the contribution of individual common variants to complex trait variation in Kosraens is typically not much greater than that observed in other populations. We show that the effects of many loci previously identified in Caucasian populations are indistinguishable in Caucasians and Kosraens, despite very different population genetics and environmental influences.

## Introduction

The use of isolated populations has a long history in genetic mapping, with benefits including founder effects, reduced genetic diversity, reduced genetic and environmental heterogeneity, and large, multi-generational pedigrees [1]–[3]. The resulting reduction in allelic heterogeneity has contributed to the success of genetic linkage and positional cloning approaches in isolated populations, particularly for the identification of Mendelian disease mutations [1]. While multiple rare mutations may segregate in an outbred population, founding events and subsequent population bottlenecks may reduce allelic diversity such that a single mutation dominates the allelic spectrum in an isolated population. In addition, previously rare mutant alleles may increase in frequency through genetic drift or natural selection, thus contributing more substantially to trait variation than in outbred populations and increasing the power of genetic mapping studies. Conceivably, the same properties that make isolated populations valuable for Mendelian trait genetics may be exploited for genome-wide association approaches to the study of complex genetic traits [3].

We have been studying the native population of Kosrae, Federated States of Micronesia, under the hypothesis that power to detect mutant alleles might be enhanced by reduced allelic heterogeneity, and that different genes (and thus biological insights) might be obtained. Our initial analyses of genotyping data from 30 Kosraen trios and ∼110,000 genome-wide SNPs showed that Kosraens exhibit strikingly reduced haplotype diversity and extended LD, likely resulting from a strong founder effect and repeated population bottlenecks [4]–[6]. These features were much more dramatic than in commonly cited “founder” populations such as Finland and Iceland. Our prior analyses, including resequencing on Kosrae, suggested that fixed marker sets such as the Affymetrix SNP genotyping products would provide better coverage for common variants in Kosraens than in any HapMap population [4].

We also previously observed that native Kosraens exhibit elevated rates of obesity and diabetes, as seen in other indigenous populations [7]–[10]. It is likely that many common mechanisms underlie the rising prevalence of obesity and metabolic disease in both Caucasian and native populations. However, given the reduced genetic diversity of isolated populations, the high prevalence of metabolic disease raises the possibilities that population-specific disease loci and fewer disease loci of relatively larger effect segregate in Kosraens.

The genetic architecture of isolated populations introduces analytic challenges which confound traditional association tests [11]. Inbreeding and the historical lack of random mating in a small population violate assumptions such as Hardy-Weinberg equilibrium which underlie many association test statistics. Members of isolated populations descend from a small number of founders, thus are related, typically in large families. In addition to “known” relationships, cryptic relatedness further confounds the test statistic, as more distant relationships may be unreported or incorrectly specified during patient interview.

We ascertained over 3,100 Kosraen adults in three screens spanning a decade and performed genome-wide association studies for 15 quantitative traits in this cohort. To do so, we developed analytic strategies to address the complexities of studying a population in which the majority of subjects are related. Our work includes: extensive validation of the extended Kosrae pedigree; identifying an analytic approach to maximize power; calibrating the association score to correct for relatedness in the cohort; and application of this method to the analysis of 15 quantitative traits. Results from the genome-wide association analyses validate our approach by detecting previously known loci for LDL-C, HDL-C, triglycerides and C-reactive protein. Additionally, our data suggest novel loci contributing to phenotypic variation in thyroid stimulating hormone (TSH) and fasting plasma glucose (FPG). While empirical power calculations suggest our study is well-powered to detect common variants of relatively large effect (≥5% variance explained) with genome-wide significance, no such effects were observed in our data with convincing statistical support.

## Results

### Sample Ascertainment

We performed a population-based screen of native Kosraens over three separate visits to the island (Table 1). The 1994 cohort was described previously [7],[12]. Self-reported family relationships were recorded for use in constructing pedigrees and blood was collected for DNA extraction and genotyping. A rich phenotypic dataset was collected for a majority of the adult population of the island, including measurements of height, weight, body mass index (BMI), waist circumference, plasma leptin, percent body fat, fasting plasma glucose, blood pressure, plasma lipids (ApoA1, HDL-C, ApoB, LDL-C, total cholesterol, triglycerides), thyroid stimulating hormone (TSH), and plasma C-reactive protein (CRP). Phenotypic data were carefully reviewed for errors in data entry, unit conversion and spurious measurements, and to verify that measurements of related traits are correlated (*e.g.*, r^2^>0.7 between BMI and waist circumference). Any values that could not be reconciled were excluded from the analysis. Heritability estimates for each trait are typically within published ranges; mean values, distribution, number of phenotyped individuals, and heritability estimates for each trait can be found in Dataset S1.

**Table 1:** Study participants successfully genotyped for the Affymetrix 500 k assay.

	N total	N unique	% Male/Female	Mean age	Median age	Age Range
**1994**	1,935	903	49.6%/50.4%	43	39	20–86
**2001**	1,968	889	40.8%/59.2%	28	24	16–80
**2003**	84	33	51.5%/48.5%	24	22	16–53
**Multiple exams**	-	1,081	35.3%/64.7%	47	46	17–89
**Total**	3,987	2,906	41.6%/58.4%	40	38	16–89

Screenings took place in 1994, 2001, and 2003. For each screen, the total and unique number of individuals examined is shown, as some participants were examined in multiple screens. For subjects examined more than once, age (years) is reported from the most recent exam.

### Genotyping

A total of 2,906 individuals were successfully genotyped using the Affymetrix 500 k mapping assay (minimum per-chip call rate 95%) (Table S1). SNPs were excluded from the analysis for the following reasons: mapping to multiple genomic locations (n = 3,462); missing >5% data (n = 43,849); or more than 10 Mendelian errors observed (n = 5,887) (Figure S1). Hardy-Weinberg equilibrium was not used as a quality filter, as it is difficult to assess in our highly related cohort using standard formulae. For the purposes of SNP quality control, allele frequencies were estimated assuming all 2,906 genotyped individuals are unrelated. After excluding monomorphic SNPs (n = 30,581), 408,775 SNPs passed technical quality filters, including 78,862 SNPs of very low frequency in Kosraens (0<MAF<0.01).

We next used data from 2,906 individuals genotyped for 400,301 polymorphic, autosomal SNPs to validate the Kosrae pedigree.

### Refining the Kosrae Pedigree with Genome-Wide Genetic Data

Genetic accuracy of the Kosrae pedigree was assessed using pairwise identity-by-descent (IBD) estimates generated in PLINK [13]. For three types of known relationships (parent-child, full sibling, and half-sibling), pairs of genotyped individuals were evaluated to determine whether estimates of the proportion IBD zero, one or two copies were consistent with the relationship reported by the patients and their families. The pedigree was corrected to reflect the true genetic relationship between pairs of individuals whose IBD estimates were inconsistent with self-reported relationships (Table 2). For example, 2,553 parent-child pairs reported by study participants were validated by genetic data, while 141 parent-child pairs were identified using IBD estimates where the relationship was previously unknown, was misreported, or not reported by study participants. In some cases, individuals were added to the pedigree as “placeholders.” For example, if genetic data indicated that one individual of a reported sibship was actually a maternal half-sibling, an ungenotyped “placeholder” was added to the pedigree as the father of the newly-discovered half-sib. Discrepancies between the genealogical and genetic pedigrees on Kosrae are not unexpected given the inherent inaccuracies of self-reported relationships, and are also consistent with known adoption practices on the island.

**Table 2:** Evaluation and refinement of the extended Kosrae pedigree using identity-by-descent estimates.

	Relationship type	Confirmed	Conflicting	Newly discovered
**Two individuals genotyped**	Parent-Child	2,553	2	141
	Full sibling	4,147	72	110
	Half sibling	351	72	271
**One individual genotyped**	Parent-Child	3,266	229	109
	Full sibling	2,384	126	33
	Half sibling	162	28	104
**No individuals genotyped**	Parent-Child	1,415	43	28
	Full sibling	941	34	21
	Half sibling	51	0	18

For each type of relationship, the number of related pairs is shown where the reported relationship and identity-by-descent estimates from genetic data were in agreement (“Confirmed”), conflicting, or added based on genetic data (“Newly discovered”). Estimates for genome-wide IBD sharing and sharing 0, 1, or 2 copies IBD were used to distinguish between the three relationship types. Individuals were added to the pedigree as necessary to represent genetic relationships, such as the addition of a “placeholder” father to reflect a newly-discovered maternal half-sib relationship. Corrections to the pedigree were made based on data from related pairs with two genotyped individuals, but impacted relationships throughout the extended pedigree.

Changes to the pedigree were made based on data from a related pair in which both individuals were genotyped. However, successive iterations of pedigree validation and correction for fully genotyped, first-degree relatives produced a “ripple effect,” also improving the accuracy of relationships involving individuals not genotyped with the 500 k assay (Table 2), and second-degree and other higher-order relationships across the extended pedigree.

After extensive comparisons with genetic data, the extended Kosrae pedigree spans eight generations and includes over 4,300 individuals (living or deceased), averaging four individuals per sibship (range 1–12). Nearly all (n = 2,900) of the subjects successfully genotyped with the Affymetrix 500 k assay can be joined in a single extended pedigree, with an additional six individuals forming three independent nuclear families. We count 58 consanguineous offspring as well as numerous marriage loops. Nearly 30% of all genotyped individuals have two genotyped parents. Fifty-six individuals appear distantly related or unrelated to any other study participants.

### Development of a Strategy for Association Analyses

Our goal was to develop an analytic framework that accommodates the complex familial relationships in the Kosraen cohort while maximizing power to detect association. We were unable to identify or develop software capable of simultaneously computing over a complex pedigree of 2,900 individuals and >330,000 SNPs. Thus, our strategy became to break the pedigree into smaller units; a similar approach was recently taken by Przeworski and colleagues in their study of recombination in the Hutterites [14]. Below we also describe the data simulation framework used to perform controlled comparisons between analytic approaches, leading to the selection of an association test. We use empirical power calculations to determine an effective sample size for our highly related cohort and estimate the power of our study across a range of effect sizes. We applied our method for association analyses in a related cohort to the study of 15 quantitative traits in native Kosraens.

### Breaking the Pedigree

We broke the extended pedigree to create smaller units that could be feasibly analyzed by existing software packages for large numbers of markers, while maximizing the number of genotyped individuals included in the analysis (as an initial, rough proxy for power) and maintaining some degree of information about relatedness between study participants. Alternative methods for breaking the pedigree were systematically explored, as described in the Materials and Methods.

**Figure 1:**
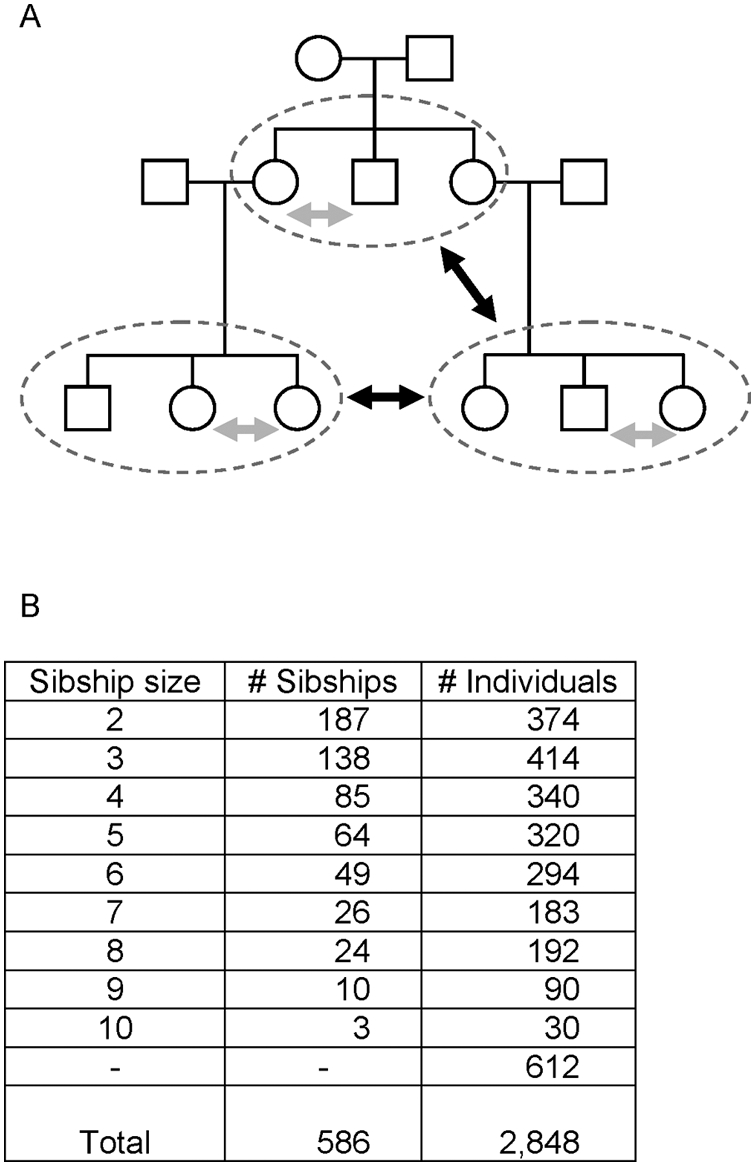
Breaking the extended Kosrae pedigree. A) The extended Kosrae pedigree is broken into sibships without parents. Parent-child or cousin relationships may exist between different sibships. Tests of association are performed within sibships (gray arrows) and between sibships (black arrows). Individuals without siblings (sibships of size 1) are filtered based on genome-wide IBD sharing to produce a maximal set of individuals with pairwise relationships equivalent to first cousins or less. Panel B shows the number of sibships of each size for n = 2,848 Kosraen individuals genotyped with the Affymetrix 500 k assay.

We selected sibships-without-parents as the unit of analysis (Figure 1A). The 2,848 non-consanguineous genotyped Kosraens were grouped into 586 sibships consisting of two or more individuals who share a mother and father (Figure 1B). Any genotyped parents are considered only in the context of the parents' sibship. Of the individuals not included in a sibship of size ≥2 (n = 612), a subset was identified in which any two members of the subset were related to the degree of first cousins or less, as determined by genome-wide IBD sharing. In the context of association analyses, this subset of individuals can be considered as sibships of size one, where relatedness between family groups is no more than first cousins.

The actual number of individuals included in the association analysis varies with the availability of phenotypic data, as individuals lacking phenotype data were omitted from the analysis of each trait. The extended Kosrae pedigree was thus broken into sibships for analysis of each of 15 quantitative traits. For example, in LDL-C, 560 sibships size ≥2 and 240 sibships of size 1 (n = 2,366 individuals total) were analyzed for association. For BMI, the analysis was limited to individuals who had reached full adult height (females age ≥22 and males age ≥24) [15], and comprised 2,073 individuals in 467 sibships of size ≥2 and 202 sibships of size 1.

Since the Kosrae cohort spans multiple generations, members of one sibship are frequently parents or cousins of other sibships. Because traditional association tests assume independence between family groups, we anticipated that relatedness between sibships would inflate the association test statistic [16],[17].

### Selection of an Association Test: Within and between Families

We used simulation to evaluate association tests and the distribution of association statistics, with the goal of selecting an association test that maximized power for our chosen family configuration of sibships-without-parents. We compared two different approaches for association analyses of quantitative traits: a within-family test vs. a combined within- and between-family test. We selected the FBAT software to represent within-family test statistics, with the expectation that it would be robust to population stratification and relatedness between families [18]. The QFAM module in PLINK includes options for within-only (PLINK/QFAM-Within) as well as within- and between-family tests (PLINK/QFAM-Total) [13]. Both options of PLINK/QFAM use permutation testing to derive empirical p-values; however, we expected the between-family test to exhibit score inflation due to known relatedness between sibships.

We used a modified simulation framework to evaluate and compare performance of the association approaches. An effect of known size was spiked into a Kosraen phenotype (BMI) and analyzed using observed Kosraen genotypes and family structure. We chose to modify an observed phenotype instead of simulating genotypes in order to preserve the complex familial correlations between genotype and phenotype on Kosrae. We selected BMI as a representative quantitative phenotype for its moderate heritability (h^2^ = 0.47 on Kosrae) and near-complete phenotyping in our cohort. Genotype data for 1,000 SNPs were randomly drawn from the larger dataset. After omitting rare SNPs (MAF<0.01), 770 SNPs remained. For each simulation, we modified the BMI phenotype to contain an association to a single SNP contributing an additional 1% to the total phenotypic variance. While this constitutes a fairly substantial single locus effect, it constitutes a small influence on the trait as a whole that does not distort the overall heritability and genome-wide relationships between genotype and phenotype. A total of 770 modified phenotypes were generated, each containing an artificial association to a different SNP in addition to the heritable and other variation in the observed BMI phenotype. Across datasets, the randomly-selected SNP associated with the spiked-in effect spanned a range of allele frequencies greater than 0.01.

Each dataset was analyzed in parallel using FBAT for a within-family association test, or using PLINK/QFAM for within-only or combined within- and between-family tests. The performance of each method was evaluated by tallying across datasets the rank of the spiked SNP within its respective dataset. The method that consistently assigned a higher rank to the spiked SNP was identified as the more powerful approach for association analyses. While genomic control is used in the actual association tests to control the false positive rate, we note that rank order is not changed by genomic control, and thus we did not employ it at this stage of evaluating methods.

**Figure 2:**
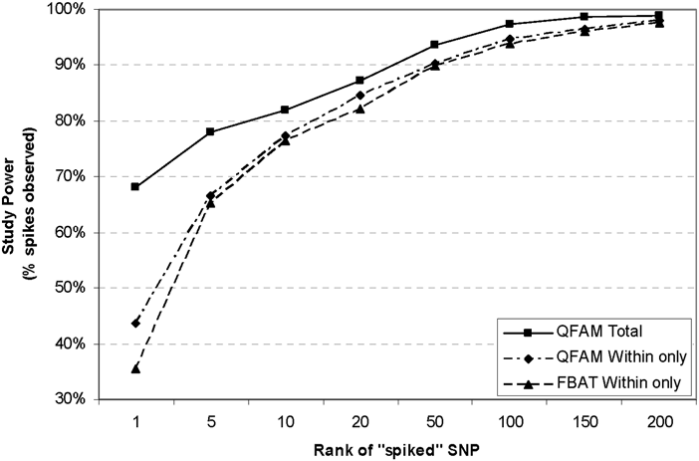
Inclusion of a between-family test of association increases study power using rank as a metric. A known effect comprising 1% of phenotypic variance explained was “spiked” into a dataset of 770 randomly selected SNPs with MAF≥0.01. Study power was evaluated for within-only (FBAT and PLINK/QFAM-Within) and within- and between-family (PLINK/QFAM-Total) tests of association. Across the 770 spiked datasets generated, study power is measured as the fraction of datasets in which the “spiked” SNP exceeds a particular rank. Ranking first out of 770 SNPs in each dataset approximates a rank of ≤440 in the context of a full genome-wide scan of ∼340,000 markers.

Comparison of the within-only vs. combined within- and between-family association test confirmed that greatest power, as measured by the rank order of the true effects, was obtained through the use of a combined within- and between-family association test (Figure 2). Within-family tests implemented in FBAT and PLINK/QFAM-Within identified the spiked SNP as the best result in 36% and 43% of all spiked datasets, respectively. A combined within- and between-family test as implemented in PLINK/QFAM-Total increased identification of the spike as the best-associated SNP to 68%. PLINK/QFAM-Total also ranked a greater proportion of spiked SNPs in the top 5 results than FBAT (78% PLINK/QFAM-Total vs. 65% FBAT), indicating that the between-family test adds substantial power to the study. In a full genome scan of ∼340,000 markers, these rank thresholds approximately correspond to the top 440 or 2,200 results, respectively, for a true effect explaining 1% of the variance.

We then examined p-value distributions for the PLINK/QFAM-Within and PLINK/QFAM-Total tests (data not shown). As expected, p-values for the within-family test follow the null distribution while the combined within- and between-family (QFAM-Total) test exhibited a systematic deviation from the null. Such inflation of the nominal association score is typical for genotyping artifacts as well as excess known or cryptic relatedness in a between-family test of association, and was anticipated from the known relatedness between sibships. We determined that the major source of score inflation in the combined within- and between-family association test (QFAM-Total) was relatedness in the cohort (Dataset S1, Figure S2). Conceptually, including closely related family units resembles population stratification, as the allele frequencies (from IBD) and phenotypes (from heritability and shared environment) are correlated in family members. In all subsequent analyses, we applied genomic control to adjust the association scores for excess relatedness [19].

**Figure 3:**
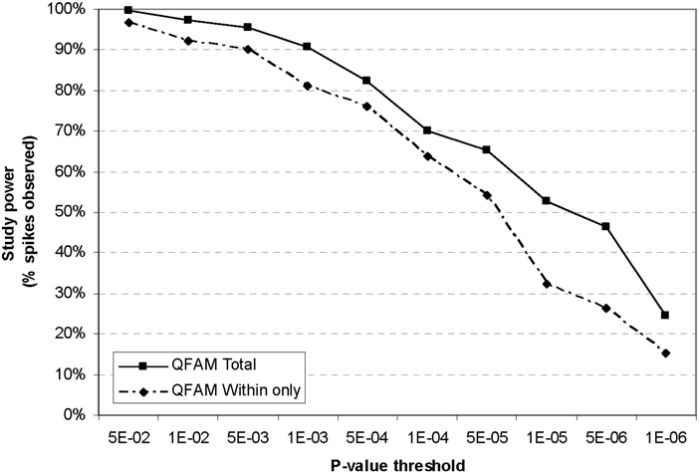
Inclusion of a between-family test of association increases study power using p-value as a metric. A known effect comprising 2% of phenotypic variance explained was “spiked” into a dataset of 770 randomly selected SNPs with MAF≥0.01. Study power was evaluated for within-only (PLINK/QFAM-Within) and within- and between-family (PLINK/QFAM-Total) tests of association. After calibrating the score distribution to the null using genomic control, study power is measured as the fraction of datasets in which the “spiked” SNP exceeds a particular p-value threshold.

After calibrating the distribution of test statistics to the null, we again evaluated the within-only vs. within- and between-family association tests using p-values to compare performance and to assess study power. Because the ability to estimate power accurately is poor when power is very low, we sought to improve power to discriminate between performances of the two tests by examining the spiked dataset containing an effect explaining 2% phenotypic variance. Using p-values as the measure of significance, we confirmed that the combined within- and between-family association test implemented in PLINK/QFAM-Total has 24.4% power compared to the within-family only test at 15.3% power to achieve an arbitrary threshold of p<10^−6^ (Figure 3).

Based on these preliminary analyses, we selected an analytic strategy as follows. The extended Kosrae pedigree is broken into smaller family units, namely sibships without parents. The remaining individuals are filtered on identity-by-descent estimates to produce a set of individuals related no more closely than first cousins, *i.e.* sibships of size one. The complete set of all sibships is filtered for each trait to include only individuals who are both successfully genotyped and phenotyped. Sibships are analyzed using a combined within- and between-family association test as implemented in PLINK/QFAM-Total, including permutation testing to correct for within-family correlation between genotype and phenotype. Finally, we compensate for relatedness between family units and any residual stratification by applying genomic control.

### Empirical Power Calculations

**Figure 4:**
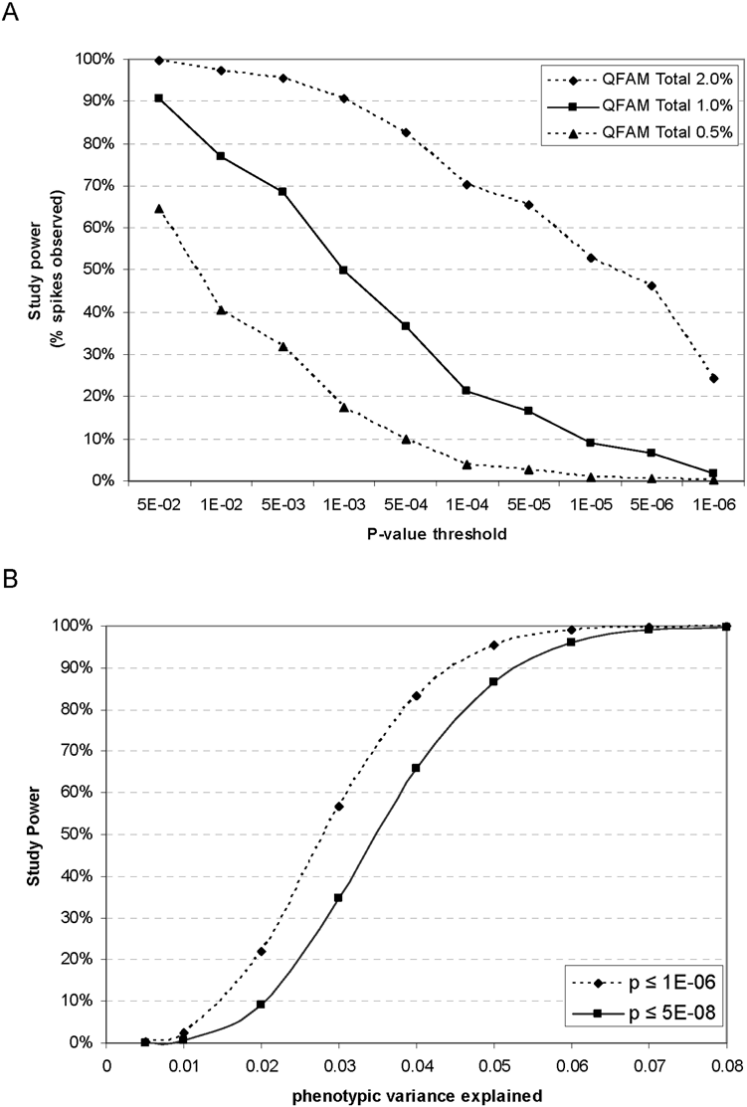
Study power over varying effect sizes. A known effect explaining 0.5%, 1% or 2% of phenotypic variance was “spiked” into a dataset of 770 randomly selected SNPs with MAF≥0.01. Study power was evaluated using a combined score from within- and between-family tests of association (QFAM-Total) with genomic control. A) Of 770 spiked datasets generated, power is measured as fraction of datasets in which the “spiked” SNP exceeds a particular p-value threshold. These data were used to estimate an effective sample size for Kosrae, from which power estimates for effects explaining up to 8% of phenotypic variance were generated (panel B).

We used the simulation data above to estimate the effective sample size for the BMI phenotype by direct observation for small effects (Figure 4A) and by extrapolation for larger effects (Figure 4B). Power from the 2,073 individuals analyzed in Kosraen sibships for BMI is comparable to that obtained from a study of 840 unrelated individuals. The more than two-fold size reduction from the actual cohort composed of sibships to an effective number of unrelated individuals highlights the excess of relatedness among our study participants.

Given the effective sample size of our cohort, we then used the Genetic Power Calculator [20] to estimate study power for effects explaining larger proportions of phenotypic variance, as our hypothesis was that such effects might exist on Kosrae (Figure 4B). Our study has ∼87% power to detect effects explaining 5% phenotypic variance at a genome-wide significant threshold of p<5×10^−8^, and >95% power to detect such effects at p<10^−6^. We concluded that our genome-wide association strategy for quantitative traits on Kosrae is well-powered to identify loci with relatively strong genetic effects, should they exist and are tagged by SNPs on the genotyping array.

### Results from the Genome-Wide Association Analyses of 15 Quantitative Traits

**Figure 5:**
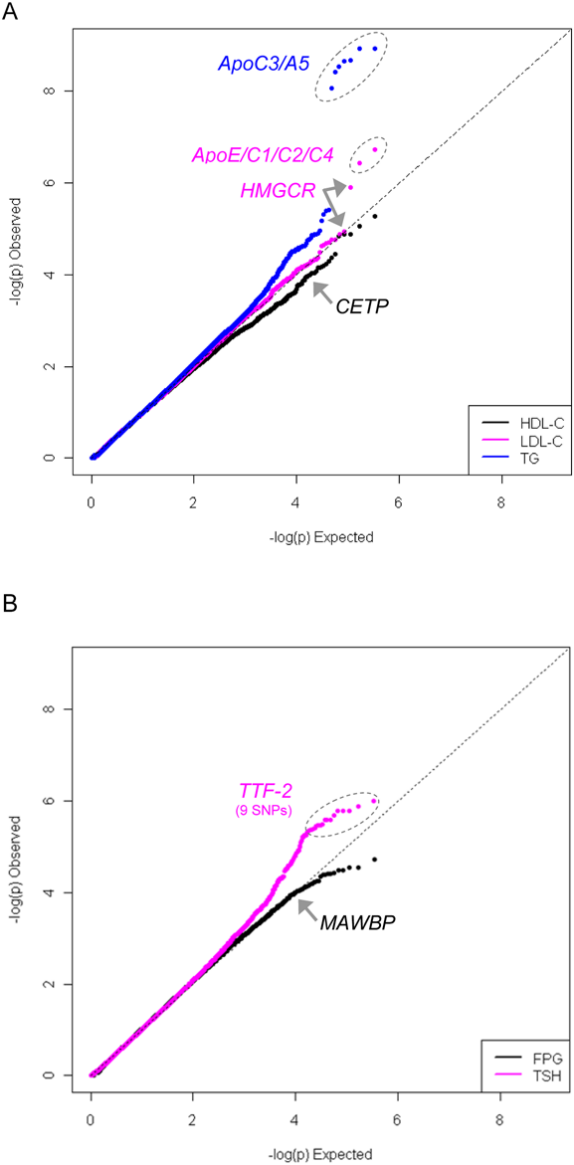
Quantile-quantile plots showing genome-wide association results for five selected quantitative traits. The extended Kosrae pedigree was broken into sibships. Association for each quantitative trait was evaluated using PLINK/QFAM-Total. Scores were adjusted for inflation due to excess relatedness using genomic control. Panel A highlights SNPs with known association to HDL-C, LDL-C and triglycerides. Panel B shows an excess of association for thyroid stimulating hormone (TSH), while association scores for fasting plasma glucose (FPG) follow the null distribution.

We applied our strategy for association analyses to the examination of 15 quantitative traits, using measurements from clinical screenings in 1994, 2001 and 2003. As anticipated from the known relatedness between sibships, scores were inflated compared to the null distribution. Score inflation ranged from λ = 1.10 for fasting plasma glucose to λ = 2.05 for HDL-C (Figure S3). Score inflation was correlated strongly with trait heritability (r^2^ = 0.42). For reference, Table 3 provides a list of SNPs with p≤10^−5^ for each trait, including genome-wide significant association (p≤5×10^−8^) between SNPs on chromosome 11 and triglycerides. Quantile-quantile (QQ) plots and the respective genomic control correction factors (λ) for select traits are shown in Figure 5; plots for all 15 quantitative traits are presented in Figure S3. The results from our genome wide scans indicate an excess of association signal over that expected by chance for LDL-C, triglycerides and thyroid stimulating hormone.

**Table 3:** Associated SNPs with p≤10^−5^ for 15 quantitative traits.

Rank	Chr	Position (Mb)	SNP	A1	A2	MAF	P-value	β	Var expl	Nearest Gene(s)
**BMI**										
1	10	1.7	rs6560749	T	G	0.14	2.0E-06	−0.32	2.4%	adarb2
**Height**										
1	7	41.3	rs10486715	A	G	0.07	3.1E-06	−0.44	2.6%	
2	7	41.3	rs17718077	C	G	0.07	3.1E-06	−0.44	2.5%	
**Waist Circumference**										
1	3	160.7	rs2222328	C	T	0.32	8.4E-07	−0.22	2.1%	schip1
2	9	22.7	rs613391	G	C	0.49	5.3E-06	0.20	1.9%	
3	9	22.6	rs527485	A	G	0.50	6.7E-06	0.19	1.8%	
4	9	22.7	rs976731	T	A	0.47	7.3E-06	0.19	1.9%	
5	9	22.7	rs976730	A	T	0.47	8.4E-06	0.19	1.8%	
**Weight**										
1	9	24.1	rs2225614	C	A	0.50	2.8E-06	−0.21	2.3%	
2	2	144.3	rs10928195	C	G	0.08	4.1E-06	−0.36	1.9%	arhgap15
3	7	137.6	rs1874326	G	A	0.47	9.0E-06	0.22	2.3%	trim24
**Leptin**										
1	3	31.5	rs882648	A	G	0.36	1.2E-05	−0.14	0.9%	ensg00000181233
**% body fat**										
1	20	44.9	rs6066084	A	G	0.06	1.5E-06	0.85	8.7%	
2	20	44.9	rs6018089	C	T	0.06	1.6E-06	0.86	9.1%	
3	20	44.9	rs6066085	C	T	0.07	2.1E-06	0.85	8.9%	
4	10	1.7	rs6560749	T	G	0.14	7.5E-06	−0.34	2.7%	adarb2
5	8	3.8	rs2554622	C	A	0.42	9.7E-06	0.23	2.5%	
**HDL-C**										
1	5	180.2	rs655601	A	C	0.47	5.3E-06	0.23	2.6%	mgat1
2	6	22.9	rs10498712	G	A	0.25	8.7E-06	0.23	2.1%	
**LDL-C**										
1	19	50.1	rs4420638	G	A	0.21	1.9E-07	0.31	3.1%	tomm40,apoc2,apoe,apoc4,apoc1
2	19	50.4	rs2159324	T	C	0.44	3.7E-07	−0.21	2.2%	mgc2650,bloc1s3,xtp7
3	5	74.7	rs3846663	T	C	0.39	1.3E-06	0.21	2.1%	col4a3bp,hmgcr
**Total Cholesterol**										
1	19	50.1	rs4420638	G	A	0.21	3.4E-07	0.28	2.5%	tomm40,apoc2,apoe,apoc4,apoc1
2	10	15.5	rs7917302	C	G	0.03	1.9E-06	−0.39	0.9%	
3	19	50.4	rs2159324	T	C	0.44	2.3E-06	−0.19	1.8%	mgc2650,bloc1s3,xtp7
4	5	74.7	rs3846663	T	C	0.39	5.5E-06	0.19	1.7%	col4a3bp,hmgcr
5	10	15.8	rs7895372	G	C	0.04	6.0E-06	−0.35	1.0%	itga8
6	7	8.8	rs17157663	A	G	0.32	8.8E-06	−0.18	1.5%	
7	7	8.8	rs16874905	T	C	0.32	9.9E-06	−0.18	1.5%	
**Triglycerides**										
1	11	116.2	rs7396835	T	C	0.32	1.2E-09	0.23	2.3%	
2	11	116.2	rs7396851	T	C	0.32	1.2E-09	0.23	2.3%	
3	11	116.2	rs2727789	G	T	0.34	2.1E-09	0.22	2.2%	
4	11	116.2	rs2071521	G	A	0.34	2.2E-09	0.21	2.0%	
5	11	116.2	rs2849176	T	C	0.34	2.9E-09	0.22	2.1%	
6	11	116.2	rs2071523	C	T	0.34	3.9E-09	0.21	2.1%	
7	11	116.2	rs6589567	C	A	0.37	8.6E-09	−0.21	2.2%	mgc13125,ensg00000110244,apoc3,apoa5,znf259
8	19	49.6	rs2722750	C	G	0.39	3.8E-06	0.16	1.3%	znf228,znf285
9	19	40.3	rs12978414	G	C	0.14	4.0E-06	0.28	1.9%	ensg00000179066,fxyd5,ensg00000126258,lgi4,fxyd3,fxyd7
10	19	50.1	rs4420638	G	A	0.21	5.0E-06	0.22	1.6%	tomm40,apoc2,apoe,apoc4,apoc1
11	4	80.9	rs10518224	A	G	0.23	6.7E-06	0.19	1.3%	
**Systolic BP**										
1	10	18.8	rs7069923	C	T	0.49	1.1E-06	0.19	1.8%	cacnb2
2	7	156.6	rs2527866	C	A	0.23	2.9E-06	−0.27	2.5%	ube3c
3	10	18.8	rs4237348	T	C	0.50	3.1E-06	0.18	1.5%	cacnb2
4	10	18.8	rs4628581	A	C	0.49	3.7E-06	0.18	1.5%	cacnb2
5	7	156.6	rs2527865	T	C	0.23	3.8E-06	−0.27	2.5%	ube3c
**Diastolic BP**										
1	5	171.0	rs254893	A	G	0.06	5.5E-06	−0.58	4.1%	
2	10	17.0	rs10508517	A	G	0.43	6.1E-06	−0.18	1.5%	cubn
**Fasting plasma glucose**										
1	10	50.2	rs10745259	T	C	0.29	1.9E-05	0.19	1.5%	c10orf71
**Thyroid Stimulating Hormone**										
1	9	97.8	rs755109	C	T	0.23	9.9E-07	−0.31	3.3%	hemgn,c9orf156
2	7	3.7	rs6462411	C	T	0.20	1.3E-06	−0.36	4.2%	sdk1
3	9	97.7	rs10983893	C	T	0.15	1.6E-06	−0.31	2.4%	
4	12	2.8	rs10848704	C	T	0.35	1.7E-06	−0.29	3.7%	ensg00000118975,fkbp4
5	9	97.7	rs4743136	G	C	0.25	1.7E-06	−0.29	3.0%	
6	7	3.7	rs10241703	T	C	0.20	2.1E-06	−0.34	3.7%	sdk1
7	7	3.7	rs6958535	C	T	0.20	2.6E-06	−0.34	3.7%	sdk1
8	9	97.6	rs925488	G	A	0.19	2.6E-06	−0.29	2.7%	
9	9	97.7	rs10983932	T	A	0.15	2.6E-06	−0.31	2.4%	
10	7	3.7	rs6959674	C	T	0.20	3.2E-06	−0.33	3.6%	sdk1
11	7	3.7	rs11514766	T	C	0.20	3.4E-06	−0.34	3.7%	sdk1
12	7	3.7	rs7804166	C	T	0.20	3.4E-06	−0.34	3.7%	sdk1
13	9	97.6	rs1877431	A	G	0.19	3.5E-06	−0.29	2.7%	
14	9	97.6	rs1588635	A	C	0.19	3.9E-06	−0.29	2.6%	
15	9	97.8	rs10984516	T	C	0.13	4.0E-06	−0.33	2.4%	hemgn,anp32b
16	7	3.7	rs1962785	C	G	0.20	4.2E-06	−0.34	3.6%	sdk1
17	9	97.6	rs2805809	A	G	0.18	4.3E-06	−0.28	2.3%	
18	7	3.6	rs12531984	G	A	0.20	4.5E-06	−0.34	3.7%	sdk1
19	7	3.7	rs10243770	C	T	0.18	4.7E-06	−0.37	4.0%	sdk1
20	9	97.7	rs10119795	C	T	0.27	4.9E-06	−0.27	2.8%	c9orf156
21	7	3.6	rs12539695	G	C	0.19	5.6E-06	−0.34	3.6%	sdk1
22	9	97.4	rs2805810	T	C	0.16	5.7E-06	−0.28	2.2%	tmod1
23	7	3.7	rs6956479	G	C	0.20	6.0E-06	−0.34	3.7%	sdk1
24	9	97.6	rs2668804	A	C	0.18	6.1E-06	−0.28	2.3%	
25	9	97.7	rs7036589	A	T	0.14	7.2E-06	−0.30	2.2%	c9orf156
26	6	98.8	rs6909430	G	A	0.08	8.1E-06	−0.49	3.8%	
27	9	97.6	rs2808693	G	A	0.18	9.6E-06	−0.28	2.3%	ensg00000188515
28	7	3.7	rs10245389	C	T	0.20	9.9E-06	−0.34	3.7%	sdk1
**C-reactive protein**										
1	19	50.1	rs4420638	G	A	0.21	1.6E-06	−0.28	2.6%	tomm40, apoc2, apoe, apoc4, apoc1
2	12	112.4	rs11066587	G	C	0.16	4.5E-06	0.26	1.8%	
3	12	119.7	rs1039302	T	C	0.36	5.2E-06	0.21	2.0%	sppl3
4	2	24.2	rs7561273	A	G	0.32	6.1E-06	−0.22	2.1%	ensg00000173957, ubxd4, fkbp1b, flj21945
5	12	119.7	rs10437838	T	A	0.35	6.2E-06	0.21	2.0%	sppl3
6	12	112.4	rs11066595	G	C	0.16	6.8E-06	0.26	1.8%	
7	2	24.0	rs17711796	C	T	0.35	7.3E-06	−0.22	2.2%	ensg00000163019
8	12	119.7	rs10431387	G	A	0.36	8.0E-06	0.21	2.0%	sppl3
9	12	119.7	rs6489780	G	C	0.35	8.4E-06	0.20	1.8%	sppl3
10	12	119.7	rs10849788	A	G	0.36	9.0E-06	0.20	1.8%	sppl3
11	2	23.9	rs2081302	A	C	0.34	9.2E-06	−0.22	2.2%	ensg00000119778, ensg00000119771
12	12	119.8	rs3809314	A	G	0.36	9.8E-06	0.20	1.8%	ensg00000174074, sppl3

A1 is the associated (minor) allele. MAF, minor allele frequency. β, effect size expressed as the number of standard deviations change in phenotype for each copy of the associated allele. “Var expl,” population phenotypic variance explained. Genes within 30 kb of the SNP are shown where applicable. For leptin and fasting plasma glucose, there were no results with p≤10^−5^; instead, the single best result is given.

We observed strong association between SNPs in previously established loci and HDL-C, LDL-C, triglyceride levels, TSH and CRP, supporting the validity of our analytic approach. For HDL-C, we observe association with rs4783962 and rs1800775 near *CETP* (p = 1.68×10^−4^ and 1.71×10^−4^, respectively), with the same allele and direction of effect as reported in Caucasian populations [21],[22]. The best-associated SNP for LDL-C is rs4420638 in the *APOE/C1/C4/C2* gene cluster on chromosome 19 (p = 1.89×10^−7^), a known locus influencing plasma levels of LDL-C and total cholesterol [23]. We also observed association between LDL-C levels and multiple SNPs in and around the gene encoding HMG-CoA reductase (*HMGCR*), the target for cholesterol-lowering statin drugs [24],[25]. Studies in Caucasian cohorts recently established this locus as a true association, with genome-wide significant p-values ∼1×10^−20^ [21]. For TSH, three SNPs (rs4704397, rs6885099, and rs2046045) previously identified in Caucasian cohorts are also associated in Kosraens, with p-values ranging from 3×10^−4^ to 1.8×10^−4^ [26]. For CRP, SNPs at the *CRP* and *HNF1A* gene loci show association and the same direction of effect on Kosrae (p = 2.0×10^−5^ and 3×10^−4^, respectively) as previously observed in a Caucasian cohort, in which the associated SNPs were either directly genotyped or are in perfect correlation (r^2^ = 1 in both HapMap CEU and ASN) [27]. The strongest association for CRP on Kosrae is with rs4420638 near the *APOE* gene (p = 1.6×10^−6^; Table 3), another previously known locus [27],[28]. This SNP is less well-correlated with the most highly-associated SNP reported in the literature (rs2075650; r^2^ = 0.37 and 0.49 in HapMap CEU and ASN, respectively) [27].

Seven SNPs near *APOC3/A5* have genome-wide significant association (p<5×10^−8^) with triglyceride levels (p = 1.2×10^−9^ to 8.6×10^−9^), including specific variants not previously implicated in Caucasian cohorts. In Kosrae, the best-associated SNP for triglyceride levels (rs7396835, p = 1.2×10^−9^) is ∼23 kb downstream of the variant recognized in Caucasians (rs2266788). Correlation between these two SNPs has not been evaluated in Kosraens, since rs2266788 is neither directly genotyped nor well-covered by other SNPs in our 500 k dataset. However, rs2266788 is uncorrelated in HapMap Asian or Caucasian samples (r^2^≤0.32 and r^2^≤0.14, respectively) with any of the seven SNPs associated with triglycerides in Kosraens. As the causal variant for this locus has not been identified, it remains to be determined whether these seven SNPs tag a causal allele common to both populations or whether independent causal variants segregate in these two ethnic groups. Besides SNPs near *APOC3/A5* and triglycerides, no other loci across all 15 traits achieved genome-wide significance (Table 3).

The most compelling evidence for a novel finding is observed in the association results for thyroid stimulating hormone (TSH). Among the top 20 results for TSH, 10 SNPs (p = 9.9×10^−7^ to 4.8×10^−6^) map to chromosome 9 at 97.6–97.8 Mb, a region encompassing the gene thyroid transcription factor 2 (*TTF-2*) (MIM 602617). Missense mutations in *TTF-2* have been reported in conjunction with thyroid agenesis and congenital hypothyroidism in humans [29]–[31]. The two best-associated SNPs for TSH, rs755109 and rs10983893, are located ∼19 kb upstream and ∼77 kb downstream of the *TTF-2* coding region, respectively. Analyses conditioning on the best-associated SNP, rs755109, suggest that a single association signal underlies association between SNPs in this region and plasma levels of TSH in Kosraens (data not shown).

In another example of association observed near a strong biological candidate, rs10998046 on chromosome 10 near *MAWBP* (MIM 612189) is modestly associated with fasting plasma glucose in Kosraens (p = 1.12×10^−4^). Upregulated expression of this gene has been reported in rat models of insulin resistance [32]. Meta-analysis of these data with publicly available results from the Diabetes Genetics Initiative [33] produces a combined p-value of 2.10×10^−6^, with 7 other neighboring SNPs producing combined p-values of 2.53×10^−6^ to 5.73×10^−6^. While extended linkage disequilibrium limits our ability to identify the causal variant in Kosraens or to exclude association to other genes in the associated region, these loci represent promising results for follow-up in other cohorts.

### Evidence for Many Variants of Small Effect Segregating on Kosrae

Our study was motivated by the hypothesis that the reduction in allelic heterogeneity resulting from the founder effect on Kosrae, combined with drift and natural selection, might produce common variants with relatively large effects segregating through the population. Empirical power calculations demonstrated that effects ≥5% should be readily detected (95% power) at p≤10^−6^. Given these power calculations, and the evidence for a series of known associated loci as described above, the consistent lack of strong association across the majority of traits argues strongly that common variants of large effect are unusual on Kosrae (Table 3), as they are in the larger populations studied to date in GWAS. The best observed p-value for each trait at a novel locus ranged from 1.9×10^−5^ for fasting plasma glucose (rs10745259) to 8.4×10^−7^ for waist circumference (rs2222328). Only two of fifteen traits, TSH and waist circumference, have strong, novel associations with p≤10^−6^. No novel associations were detected in any trait with p<8×10^−7^. Interestingly, these data indicate that on Kosrae, common variants in LD with SNPs on the genotyping array are of small effect in this founder population, similar to the genetic architecture observed in Caucasian populations.

If the effect sizes for common variants are similar in Kosraens and Europeans, then the lack of strong associations is unsurprising given the modest size of our cohort. We observe modest evidence for SNPs near multiple other loci which have been convincingly replicated in other cohorts [21],[22],[27],[33],[34], including HDL-C and *LPL* (p = 0.025, rs17411024) or *LIPC* (p = 4.5×10^−3^, rs11071386); triglycerides and *GCKR* (p = 0.015, rs780094), and LDL-C and *APOB* (1.6×10^−3^, rs7575840). These and other common variants of small effect identified in Caucasian populations may also influence trait variation on Kosrae, despite lack of genome-wide statistical significance for association.

### Comparison of Effects and Allele Frequencies in Caucasians and Kosraens for Known Associated Loci

We examined data for previously associated SNPs not under the null hypothesis of no effect on trait value, but rather under the alternative hypothesis that the effect seen in Europeans was also observed on Kosrae. We compared the effect sizes (β-coefficients) and allele frequencies for known loci in Caucasians to those observed in our study. Specifically, we identified 45 established associations for BMI, height, lipids, fasting plasma glucose, TSH and CRP (Table 4) where the best-associated SNP in the literature was directly genotyped in Kosraens or had a strong proxy (r^2^≥0.95) in HapMap Caucasians and Asians [18]–[22], [34]–[36]. A test of heterogeneity for the magnitude and direction of β-coefficients was performed for 39 SNPs with MAF≥0.05. Six SNPs were omitted from the comparison of effect sizes, as there is little power to estimate the individual effects for SNPs observed at very low frequencies.

**Table 4:** Association results on Kosrae for select previously known, associated loci.

				Caucasian				Kosraen				
SNP	Study	Nearest Gene(s)	Allele	Freq	P-value	β	on 500 k	Freq	P-value	β	P_het_ β	P_het_ Freq
**BMI**												
rs9939609	1	FTO	A	0.45	2.0E-20	0.10	same	0.12	0.10	0.14	0.65	0.16
rs17782313	2	MC4R	C	0.28	2.8E-15	0.05	same	0.08	0.55	−0.06	0.26	0.42
**Height**												
rs6763931	3	ZBTB38	A	0.48	1.4E-27	0.07	rs6440003	0.25	0.16	0.08	0.88	−
rs724016	4	ZBTB38	G	0.48	8.3E-22	0.37	rs6440003	0.25	0.16	0.08	2.8E-06	−
rs1042725	4	HMGA2	T	0.49	2.7E-20	−0.48	same	0.55	0.70	0.01	5.5E-42	0.84
rs6060369	4	GDF5-UQCC	C	0.36	1.4E-16	0.44	same	0.31	0.51	0.05	5.2E-08	0.85
rs798544	3	GNA12	G	0.72	6.5E-15	0.06	same	0.47	0.18	0.08	0.68	0.18
rs3748069	3	GPR126	A	0.73	4.5E-14	0.07	rs7755109	0.25	0.36	0.07	0.92	−
rs1812175	3	HHIP	C	0.81	9.7E-12	0.08	same	0.63	0.05	0.11	0.60	0.48
rs7153027	3	TRIP11, FBLN5, ATXN3, CPSF2	A	0.61	1.1E-10	0.06	same	0.77	0.46	−0.06	0.14	0.47
rs6830062	3	LCORL, NCAPG	T	0.84	1.3E-10	0.06	same	0.94	0.86	0.03	0.80	0.60
rs3760318	3	CRLF3, ATAD5, CENTA2, RNF135	C	0.64	1.8E-09	0.06	rs7225461	0.56	0.31	0.07	0.90	−
rs2282978	3	CDK6, PEX1, GATAD1, ERVWE1	C	0.37	9.8E-09	0.06	same	0.06	0.61	−0.07	0.33	0.10
rs967417	3	BMP2	C	0.57	1.5E-08	0.04	same	0.13	0.90	0.03	0.97	0.05
rs4743034	3	ZNF462	A	0.24	2.1E-08	0.05	same	0.14	0.41	−0.10	0.21	0.71
rs678962	3	DNM3	G	0.16	3.2E-08	0.05	rs12411264	0.14	0.77	−0.01	0.06	−
rs4533267	3	ADAMTS17	A	0.28	3.3E-08	0.06	same	0.40	0.19	0.07	0.76	0.51
rs7846385	3	PXMP3, ZFHX4	C	0.34	4.7E-08	0.05	same	0.16	1.00	−0.01	1.00	0.26
rs2562784	4	SH3GL3-ADAMTSL3	G	0.17	6.4E-08	0.34	same	0.42	0.20	−0.08	2.9E-11	0.15
rs4794665	3	NOG, DGKE, TRIM25, COIL, RISK	A	0.53	9.9E-08	0.04	same	0.26	0.65	−0.04	0.40	0.33
**HDL-C**												
rs1800775	5	CETP	C	0.43	1.0E-73	−0.18	same	0.58	1.7E-04	−0.19	0.86	0.55
rs4846914	5	GALNT2	G	0.42	2.0E-13	−0.07	same	0.58	0.58	−0.03	0.34	0.54
rs2156552	5	LIPG, ACAA2	A	0.20	2.0E-07	−0.07	same	0.03	0.45	0.10	−	0.24
rs3890182	5	ABCA1	A	0.09	3.0E-10	−0.10	same	0.37	0.18	0.07	6.8E-04	0.12
rs328	5	LPL	G	0.13	9.0E-23	0.17	rs10503669	0.98	0.04	−0.38	−	−
**LDL-C**												
rs12654264	5	HMGCR	T	0.42	1.0E-20	0.10	same	0.42	2.4E-05	0.18	0.07	1.00
rs693	5	APOB	A	0.49	1.0E-21	0.12	same	0.09	0.15	0.11	0.88	0.05
rs4420638	5	APOE cluster	G	0.18	1.0E-60	0.19	same	0.21	1.9E-07	0.30	0.05	0.92
rs16996148	5	CILP2,PBX4	T	0.06	3.0E-08	−0.10	same	0.17	0.58	−0.03	0.11	0.66
**Triglycerides**												
rs328	5	LPL	G	0.13	2.0E-28	−0.19	rs10503669	0.98	0.53	0.10	−	−
rs693	5	APOB	A	0.49	2.0E-07	0.08	same	0.09	0.17	0.10	0.81	0.05
rs12130333	5	ANGPTL3, DOCK7,ATG4C	T	0.24	2.0E-08	−0.11	same	0.04	0.87	0.02	−	0.44
rs780094	5	GCKR	T	0.38	3.0E-14	0.13	same	0.27	1.5E-02	0.12	0.88	0.68
rs17321515	5	TRIB1	G	0.40	4.0E-17	−0.08	same	0.70	1.3E-02	−0.11	0.47	0.20
rs16996148	5	CILP2,PBX4	T	0.06	4.0E-09	−0.10	same	0.17	1.6E-03	−0.16	0.25	0.66
rs4846914	5	GALNT2	G	0.42	7.0E-15	0.08	same	0.58	1.6E-03	−0.16	2.0E-06	0.54
rs17145738	5	BCL7B,TBL2,MLXIPL	T	0.12	7.0E-22	−0.14	same	0.03	0.79	−0.02	−	0.73
**Fasting plasma glucose**												
rs563694	6	G6PC2	A	0.65	6.4E-33	n/a	same	0.95	0.28	0.21	−	0.13
rs1799884	7	GCK	A	0.20	1.0E-09	0.06	same	0.14	0.06	0.15	0.26	0.82
**Thyroid Stimulating Hormone**												
rs4704397	8	PDE8B	A	0.48	1.3E-11	0.21	same	0.77	3.0E-04	0.25	0.60	0.28
**C-Reactive Protein**												
rs7553007	9	CRP	A	0.33	2.2E-26	−0.20	same	0.38	0.03	−0.11	0.06	0.86
rs1892534	9	LEPR	A	0.38	6.5E-21	−0.17	same	0.82	0.13	−0.09	0.18	0.03
rs7310409	9	HNF1A	A	0.39	6.8E-17	−0.15	rs2393791	0.44	3E-04	−0.16	0.82	−
rs780094	9	GCKR	A	0.41	6.7E-15	0.14	same	0.73	0.99	7E-4	0.01	0.12
rs4129267	9	IL6R	A	0.40	2.0E-8	−0.10	rs4537545	0.36	0.02	−0.10	1.00	−

1 Frayling *et al.* (2007) Science 316:889.

2 Loos *et al.* (2008) Nat Genet 40:768.

3 Gudbjartsson *et al.* (2008) Nat Genet 40:609.

4 Lettre *et al.* (2008) Nat Genet 40:584.

5 Kathiresan *et al.* (2008) Nat Genet 40:189.

6 Chen *et al.* (2008) J Clin Invest 118:2620.

7 Weedon *et al.* (2005) Diabetes 54:576.

8 Arnaud-Lopez *et al.* (2008) Am J Hum Genet 82:12.

9 Ridker *et al.* (2008) Am J Hum Genet 82:1185.

For SNPs not directly genotyped on the Affymetrix array (n = 9), association results are reported for a proxy on the Affymetrix chip with strong correlation (r^2^≥0.95) to the original SNP in both HapMap Caucasian and Asian populations. The effect size (β) is expressed as the number of standard deviations change in phenotype for each copy of the associated allele. “P_het_ β” denotes p-values for the test of heterogeneity between the Caucasian and Kosrae effect sizes. SNPs with low frequency in Kosraens (MAF<0.05; n = 6) were omitted from the test of heterogeneity for effect sizes. “P_het_ Freq”denotes p-values for similarity between frequency of the risk allele in Caucasians and Kosraens. SNPs not directly genotyped on the Affymetrix array were omitted from the comparison of allele frequencies. “−,” not analyzed.

Of the 39 SNPs examined for effect sizes on Kosrae, only 6 loci had significantly different (p≤0.01) effects in Caucasians and Kosraens (p = 5.5×10^−42^ to 6.8×10^−4^), of which 4 SNPs were associated with height in Caucasians. Over 70% of the loci evaluated (n = 28) had effects which were of indistinguishable magnitude and direction (p≥0.1) in the two populations.

We next considered whether differences in allele frequency could underlie the lack of association in Kosraens to loci with strong support in European studies. Of 45 established associations (Table 4), allele frequencies were compared for 36 SNPs directly typed on the Affymetrix array. For each risk allele, we identified SNPs on the Affymetrix array with frequencies in HapMap CEU within 2% of the risk allele frequency in HapMap CEU. For that set of SNPs, we generated a distribution of allele frequency differences between HapMap CEU and Kosrae. To determine whether a risk allele has an unusual difference in frequency between Kosraen and HapMap CEU, we examined the difference in frequency for the risk allele in the context of the complete distribution of allele frequencies. Over 85% of the SNPs evaluated (n = 32) have statistically indistinguishable frequencies in Kosraens and Europeans (empirical p≥0.1) while none of the loci examined had significantly different (empirical p≤0.01) frequencies in the two populations.

Together, these data suggest that Kosraens segregate many of the common variants that have been identified in Caucasian populations, and that effect sizes for a majority of those variants on Kosrae is not detectably different from that observed in Caucasians despite a dramatically different population history and environment. The empirical similarity of these genetic architectures lends support to the concept of combining association studies across populations to take advantage of neutrally arising differences in allele frequency and LD patterns to aid in confirmation and fine mapping of common disease variants.

## Discussion

We describe genome-wide association analyses in a population-based cohort with extensive family structure, and explore the value of genetic studies in a population isolate with high levels of linkage disequilibrium and relative allelic homogeneity [4]. Our goal was to take advantage of the population genetic features of this isolated population while maximizing the power to detect associations. We broke the extended Kosrae pedigree into sibships to create a computationally tractable dataset that uses as many genotyped individuals as possible. Empirical power calculations show that testing for association both within and between sibships gives more power than within-family tests alone. We used permutation testing and genomic control to correct for score inflation. Association to true biological variants was clearly observed for several known lipid loci, including *APOE*, *CETP*, *HMGCR* and *APOC3/A5*. Our ability to detect multiple loci with known association indicates that our analytic strategy is adequate to identify true disease loci.

Suggestive evidence for association was observed for thyroid stimulating factor (TSH) to SNPs in the gene encoding thyroid transcription factor 2 (*TTF-2*), a strong biological candidate with no previously known association. Associations near *APOC3/A5* for triglycerides and near *TTF-2* for TSH also highlight the possibility of island-specific variants or differences in LD between Kosraens and Caucasians that may be useful in identifying causal variants common to both populations.

Our study tests the hypothesis that reduced genetic diversity, genetic drift and/or natural selection might have resulted in a class of common alleles with large effects on metabolic phenotypes. Reduced diversity is evident in our study and consistent with our earlier observations [4], with 20% of the SNPs (n>109,000) passing technical quality filters having minor allele frequencies <0.01 in Kosraens. Empirical estimates of study power showed that we have 95% power to observe effects explaining ≥5% phenotypic variance at p<10^−6^. And yet, no large effects of this sort were detected. This is similar to the pattern observed in other populations, where the majority of common variants have individually modest effects, typically explaining ≤2% of phenotypic variance [21], [22], [35]–[37]. Our genome-wide data expand on and confirm previous work suggesting that many genes of small effect influence trait variation in both outbred and founder populations such as Kosrae [38].

While our cohort encompasses ∼65% of the adult population on Kosrae, limited sample size, coupled with substantial relatedness between study participants, reduces the power of our study in comparison to recently published genome-wide association studies and meta-analyses for common diseases. It is interesting, given the widespread and reasonable predictions that gene-by-gene and gene-by-environment effects modulate marginal associations, that a comparison of allele frequencies and the direction/magnitude of effects for loci originally identified in Caucasian cohorts shows that a majority with statistically indistinguishable effects in Kosraens.

We also note that the set of biologically relevant variants influencing metabolic traits is unlikely to be wholly identical between Kosraens and Caucasians. For example, heritability of total plasma cholesterol is similar in Kosraens and Caucasians, but the population mean is approximately 20 mg/dL lower in Kosraens. Variants specific to Kosraens may underlie the phenotypic difference between populations; these variants may lie in novel genes or genes previously implicated in disease or trait variation. Identification of such variants in Kosraens and other ethnic groups may shed light on biological pathways and aspects of disease biology that might otherwise be overlooked in purely Caucasian studies.

Validation of any novel association results in our study is hampered by the lack of genome-wide scans in cohorts with an ethnic origin and population history similar to Kosrae. The majority of studies to date have been performed in Caucasians. Further work is required to assess in Kosraens the extent of genetic drift and selection under strikingly different environmental pressures. Replication of true “island” variants would likely be difficult or impossible in existing Caucasian cohorts and underscores the need for the inclusion of diverse ethnic groups in genetic studies.

It is also worth noting that although extended LD on Kosrae facilitates locus identification through greater genome coverage, it hampers fine-mapping efforts. In the event that novel association results can be validated or replicated in other populations, a comparison of LD patterns between populations will likely be important to identify the causal variant.

While future methods will no doubt improve on our analytical approach, we describe approaches which may be useful to others undertaking genetic studies in population isolates. Tools for validating pedigrees with genetic data greatly facilitated the review of millions of pairwise IBD estimates, highlighted inconsistencies in the reported pedigree, and assisted in the identification of previously unknown first-degree relationships. We show that applying a combined within- and between-family test of association to the subunits of a large extended pedigree increases study power. In addition, the simulation framework we describe for empirical power calculations will be useful for evaluating and comparing the performance of other methods for association analyses as they become available.

The current analysis assesses the role of common variants influencing phenotype on the island of Kosrae, but does not evaluate the role of rare variants. In fact, the analytic challenges posed by extensive relatedness in this cohort and the previously demonstrated extended LD in the population suggest that Kosraens may be particularly informative for other analytic methods such as homozygosity mapping. Recent, severe population bottlenecks and subsequent rapid expansion have greatly enriched Kosraens for long stretches of homozygosity. These homozygous segments act as proxies for rare recessive variants segregating in the population and are predicted to greatly increase our power to detect such variants. We are currently developing methods for homozygosity mapping in this unique population. We anticipate that homozygosity approaches for the detection of rare, recessive alleles, coupled with direct sequencing studies to characterize variation on Kosrae not captured by existing genotyping platforms, will complement the association studies for common variants presented here. Together, these three approaches will provide a more complete picture of genetic variation in population isolates, and the underlying role of drift and natural selection on the architecture of metabolic traits on Kosrae.

## Materials and Methods

### Ethics Statement

The study was approved by the Institutional Review Boards at all participating institutions, including Rockefeller University (protocol #JFN-0282-0707), Massachusetts Institute of Technology (COUHES protocol #0602110607) and Massachusetts General Hospital (protocol #2006-P-000211/6; MGH). All patients provided written informed consent (in English or Kosraen) for the collection of samples and subsequent analysis.

### Sample Collection

During screenings performed in 1994, 2001 and 2003, patients were recruited by public announcements and came to the clinic following an overnight fast. The 1994 screen was described previously [7],[12]. Briefly, informed consent was obtained from each patient (forms available in Kosraen), along with self-reported information on identity of family members, medical history, current medications, lifestyle, diet, exercise, and ethnicity. Blood was collected from Kosraens in the fasted state and centrifuged. Plasma and buffy coats were frozen and shipped to Rockefeller University for serological assays and DNA extraction, respectively. IRB approval was obtained from all participating institutions.

### Clinical Data

Quantitative trait measurements were log- or square root- transformed to approach normality, adjusted for age and gender where applicable, and converted to Z-scores. An average Z-score was used for patients screened in multiple collection years or monozygotic twins. Individual trait descriptions are available in the Supplemental materials (Dataset S1).

Genotypes were analyzed for association with fifteen quantitative traits: body mass index (BMI), height, weight, waist circumference, plasma leptin, percent body fat, diastolic and systolic blood pressure, fasting plasma glucose, thyroid stimulating hormone, HDL-C, LDL-C, total plasma cholesterol, triglycerides and high-sensitivity plasma C-reactive protein.

### Genotyping

Data from the Affymetrix 500 k assay were generated at Affymetrix, South San Francisco, CA. Genotypes were called with the BRLMM algorithm. A minimum call rate of 95% was required for each chip (Table S1). The two chips in the 500 k assay (enzyme fractions Sty and Nsp) were matched by genotype concordance and gender concordance between each chip and the clinical data for that sample. Of the ∼3,100 subjects ascertained, 2,906 individuals were successfully genotyped according to these criteria. Per-SNP quality filters included: mapping to a unique genomic location, minimum per-SNP call rate of 95%, fewer than 10 Mendelian errors, and minor allele frequency (MAF) >0. 408,775 SNPs met these criteria. For the purposes of SNP quality control, allele frequencies were estimated assuming all 2,906 genotyped individuals were unrelated. Hardy-Weinberg equilibrium was not used as a quality filter, as it cannot be assessed by standard formulae in our highly related cohort.

Autosomal SNPs with MAF≥0.01 were analyzed for association with each trait, where MAF was calculated using the individuals phenotyped for that trait. Sibling relationships were accounted for according to default procedures in PLINK. The number of SNPs analyzed ranged from 332,890 (TSH) to 345,026 (Height).

### Pedigree

Study participants provided names and birthdates of their relatives during the patient interview. Information from multiple patient records was cross-referenced and used to reconstruct extended pedigrees. Relationships reported by subjects in the 1994 screen were validated genetically using Mendelian inheritance checks and identity-by-state analyses with microsatellite markers and the pedigree was modified to reflect the genetically accurate relationships [12],[39]. Subjects screened in 2001 and 2003 were originally incorporated into the pedigree on the basis of genealogical information.

SNP genotyping data were subjected to identity-by-descent estimation using PLINK [13]. Thresholds of IBD sharing for parent-child, full-sibling, and half-sibling relationships were empirically determined from the distribution of genome-wide IBD scores for known relationships. We used empirical ratios of total sharing and the proportion of genome shared in 0, 1, or 2 copies between two individuals to evaluate whether genetic evidence supported putative relationships reported in the Kosrae pedigree. A complete list of putative first-degree relative pairs (parent-child, sibling, half-sibling) was extracted from the pedigree. For each putative first-degree relative pair, we examined genetic evidence supporting or refuting that relationship and corrected relationships in the Kosrae pedigree accordingly. For example, in a set of individuals forming a putative nuclear family, we verified that all combinations of parent-child relationships and sibling relationships met our criteria for genetic relatedness. “Placeholder” individuals were added to the pedigree as necessary to reflect genetic relationships, such as the addition of a “placeholder” father for a newly-discovered maternal half-sibling. The correction of numerous relationship pairs and our ability to detect cryptic relationships enabled consolidation or elimination of over 70 non-genotyped ancestors, resulting in “tightening” of the Kosrae family tree.

Thirteen pairs of monozygotic twins and thirty-five duplicate sample pairs were identified by genotype similarity. Sample identity was confirmed from patient records (name, birth date) and one subject from each pair was included in the association analyses.

We identified 58 offspring from consanguineous matings, where a common ancestor could be identified in the extended Kosrae pedigree; these consanguineous offspring were excluded from association analyses. An additional nine individuals were excluded from the dataset, as they self-reported non-Micronesian ethnicities and could not be connected to the pedigree.

### Breaking the Pedigree

Three approaches were considered to break the extended pedigree into smaller units.

*Founders* consist of a filtered subset of sibships-without-parents, such that any genotyped offspring of a founder sibship are removed from the dataset. Founder sibships are drawn primarily from the upper levels of the Kosrae family tree. For example, 582 “founders” in 247 sibships were identified for the BMI phenotype.

*Sibships without parents* consist of two or more individuals known to share both parents. Since the Kosrae cohort spans multiple generations, members of one family group are frequently parents or cousins of other family groups. Information about parents is used to define a sibship; however, any genotyped parents are considered only in the context of their own siblings. For BMI, 1,871 individuals were included in 467 sibships of size ≥2.

*Nuclear families* consist of two genotyped parents and one or more offspring. Where one genotyped parent is available, offspring are included as a sibship without parents and the genotyped parent is included in the context of its own siblings. Where no genotyped parents are available, individuals contribute as members of a sibship without parents.

#### “Sibships of size one” and “Unrelateds”

Genome-wide estimates of identity-by-descent (pihat) were used to select a subset of distantly related individuals. Genotyped individuals were randomly ordered, and individuals were selected if they were related to every other member of the group below a set threshold. A threshold of pihat ≤0.125 was used for individuals included in the association analyses (“sibships of size one”), corresponding to a relationship of first cousin or less. This selection process was repeated for 1,000 iterations, after which the largest set of individuals was identified for further use. These individuals contribute in the Between-family association score. For example, 202 “sibships of size one” contribute to the analysis for BMI.

A more stringent threshold of pihat ≤0.08, applied to the entire dataset of 2,906 study participants, resulted in n = 133 individuals related as less than first cousins. These individuals are treated as independent observations (“unrelateds”), suitable for use in any analysis requiring unrelated individuals (*e.g.*, calculating allele frequencies, LD).

#### Selection of a scheme to break the extended pedigree

In selecting a method to break the extended Kosrae pedigree, we considered three factors: maximum use of genotyped individuals (a rough proxy for power); minimal inflation of the association test statistic; and the practical consideration of retaining similar family structures across multiple traits.

“Founders” (n = 582) have the fewest relationships with other individuals in the complete pedigree and were expected to minimize association score inflation due to excess relatedness between families. However, the exclusion of over 2,300 genotyped individuals from the analysis and concomitant loss of power persuaded us against this family configuration.

Sibships-without-parents and nuclear families include similar numbers of individuals for a given trait. We note that the optimal configuration of nuclear families varies across phenotypes, whereas sibships-without-parents minimize differences in family structure across multiple traits. Individuals lacking a phenotype are simply omitted from a sibship and do not radically change the number of sibships available for analysis. The extended Kosrae pedigree was broken into sibships-without-parents separately for each trait and analyzed for association.

### “Spiked” Datasets for Data Simulations

We performed empirical evaluations of power for each association method using simulated datasets. We “spiked” an effect of known size (explaining an additional 0.5%, 1% or 2% variance) into an existing Kosraen phenotype (BMI) and analyzed this modified phenotype with observed Kosraen genotypes, thereby retaining the true genotype-phenotype correlation between related study subjects. A subset of 1,000 SNPs across the genome were randomly selected and filtered to retain SNPs with MAF>0.01. The remaining 770 SNPs were analyzed for association with the spiked phenotype. A total of 770 spiked phenotypes were generated, in which each phenotype was altered to reflect association to a different SNP of the random subset. These 770 phenotypic datasets were analyzed for association to the spiked SNP using FBAT and PLINK/QFAM under an additive model [13],[40].

### Calculating Effective Sample Size

We used empirical power estimates from the BMI “spiking” experiment and the module for variance components QTL association for sibships in the Genetic Power Calculator [20] to estimate the effective sample size of our cohort, or the number of unrelated individuals required to obtain power equivalent to that provided by the Kosraen sibships. Power calculations were performed assuming no dominance, minor allele frequency of 0.2, and direct genotyping of the causal variant. For BMI, the 2,073 individuals included in the association analysis have power equivalent to ∼840 unrelated individuals.

### Association Analyses

Quantitative trait data were analyzed under an additive model using the QFAM module of PLINK [13]. Nominal scores were permuted to obtain an empirical p-value while maintaining familial correlation between genotype and phenotype. The permutation procedure employed by QFAM corrects for relatedness within families. Between-family relatedness is not addressed in QFAM and is the major source of score inflation (see Dataset S1, Figure S2). Genomic control was used to correct for score inflation introduced by relatedness between family units (sibships) [19].

We account for multiple testing by assuming a threshold of p≤5×10^−8^ for genome-wide significance, following the work of Pe'er *et al* (2008) and Dudbridge and Gusnanto (2008) [41],[42]. This approach assumes approximately 10^6^ independent tests across the genome and requires an additional p≤0.05. This significance threshold is likely conservative on Kosrae, where the true number of independent tests is likely to be smaller because of the extended LD, and so alleviates the multiple testing burden.

### Comparison of Allele Frequencies for Known Associated Loci

Association results for known, associated loci were drawn from studies in large Caucasian cohorts for multiple traits. For each Caucasian risk allele where the SNP was directly genotyped in Kosraens, we determined its frequency in HapMap CEU. We identified SNPs on the Affymetrix array that have frequencies in HapMap CEU within 2% of the frequency of the risk allele in HapMap CEU. For each SNP in that set, we calculated the difference in allele frequency between HapMap CEU and Kosrae. These values were used to generate an empirical distribution of allele frequency differences. For the risk allele, we calculate the difference in allele frequency between HapMap CEU and Kosrae, and place this difference on the empirical distribution to determine significance.

### Comparison of Effect Sizes for Known Associated Loci

Association results for known, associated loci were drawn from studies in large Caucasian cohorts for multiple traits. Caucasian loci were limited to SNPs directly genotyped in Kosraens, or where a strong proxy was genotyped in Kosraens (r2≥0.95 in HapMap CEU and ASN). SNPs with MAF<0.05 on Kosrae were omitted from comparison, as power is low to estimate effect sizes accurately for rare SNPs. We assumed the Caucasian β estimates for each of the traits to be a fixed value. A test of heterogeneity for the magnitude and direction of the effect in Caucasians and Kosraens was performed as follows [43]: 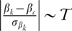 Where β_k_ and β_c_ are the effect sizes for Kosrae and Caucasian populations, 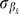
is the standard deviation on the β_k_, and is distributed like a *T*.

## Supporting Information

Figure S1 Marker quality control for SNPs in the Affymetrix 500 k assay.
(0.01 MB PDF)

Figure S2 Excess relatedness is the major source of association score inflation. Association score inflation was evaluated in three subsets of the Kosrae cohort using the BMI phenotype. Score inflation is greatly reduced in “unrelated” (less than first cousins) individuals and in a set of sibships filtered to remove all parent-child relationships between sibships, as compared to all available sibships in the cohort.
(0.06 MB PDF)

Figure S3 Quantile-quantile plots showing genome-wide association results for 15 quantitative traits. For each trait, the number of individuals used in the analysis, heritability, and genomic control correction factor (lambda) are given.
(0.17 MB PDF)

Table S1 Genotyping statistics for the Affymetrix 500 k assay.
(0.04 MB DOC)

Dataset S1 Supplemental Trait Descriptions.
(0.81 MB DOC)
