# Supplementary figures and images for: Genome-Wide Association Studies in an Isolated Founder Population from the Pacific Island of Kosrae
Source: PLoS Genet. 2009 Feb 6;5(2):e1000365. doi: 10.1371/journal.pgen.1000365 (PMC2628735; doi:10.1371/journal.pgen.1000365)

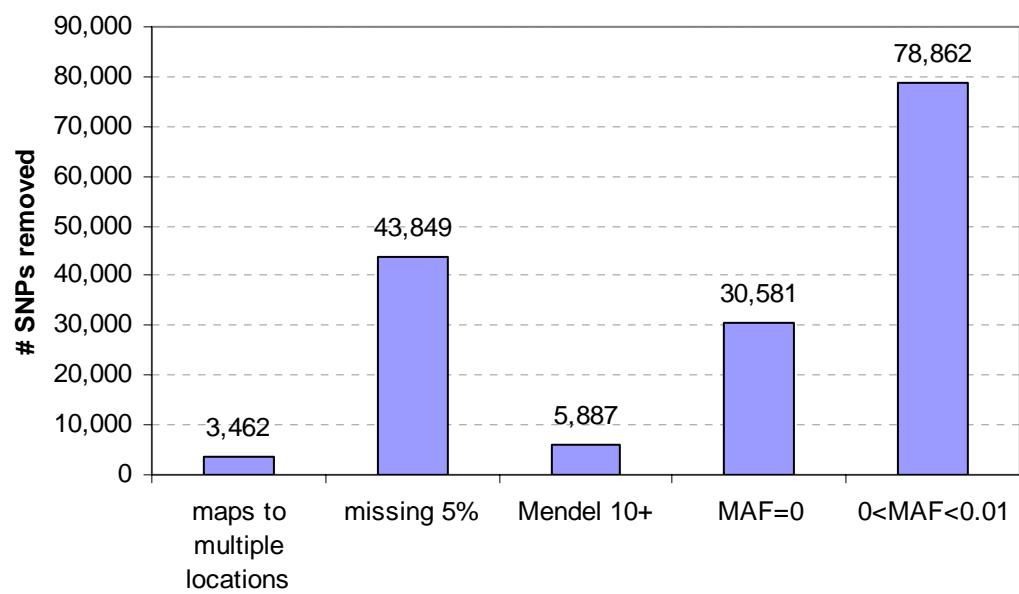

Supplement: Figure S1 — Marker quality control for SNPs in the Affymetrix 500 k assay. (0.01 MB PDF) [file pgen.1000365.s001.pdf]
