# Supplementary material for: Genome-Wide Association Studies in an Isolated Founder Population from the Pacific Island of Kosrae
Source: PLoS Genet. 2009 Feb 6;5(2):e1000365. doi: 10.1371/journal.pgen.1000365 (PMC2628735; doi:10.1371/journal.pgen.1000365)
Supplement: Figure S2 — Excess relatedness is the major source of association score inflation. Association score inflation was evaluated in three subsets of the Kosrae cohort using the BMI phenotype. Score inflation is greatly reduced in “unrelated” (less than first cousins) individuals and in a set of sibships filtered to remove all parent-child relationships between sibships, as compared to all available sibships in the cohort. (0.06 MB PDF) [file pgen.1000365.s002.pdf]

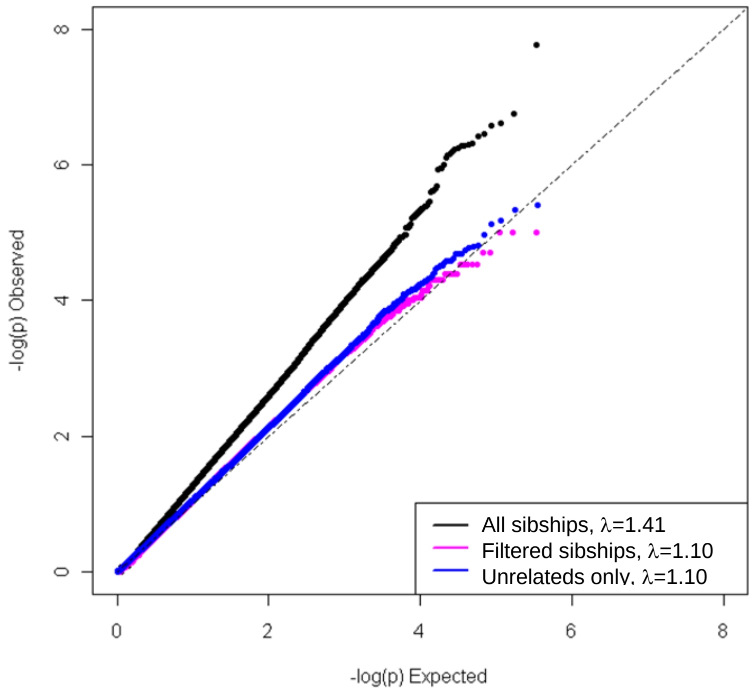

Excess relatedness is the major source of inflation in the association test statistic

We investigated the source of score inflation in our association test using the observed (not modified/spiked) BMI phenotype. Using PLINK/QFAM-Total with permutation testing, the sibships-without-parents dataset produced genome-wide association results for BMI with a genomic control inflation factor (λ) of 1.41 (Figure S2). To determine the impact of genotyping artifact on the score distribution, we analyzed a subset of 88,431 common SNPs with 100% genotyping completeness. Score inflation was unchanged from the larger marker set, suggesting that genotyping artifact is not a major cause of score inflation (data not shown). This observation is consistent with the null score distribution observed for the within-family test of association using the full marker set.

To examine the impact of excess relatedness on the score distribution, we filtered individuals to reduce relatedness between family units. Removing sibships containing parents of another sibship substantially reduced score inflation (λ= 1.10) as compared to using all sibships (λ=1.41) (Figure S2). A set of "unrelated" individuals (n=133; related less than first cousins)

produced a score distribution with λ=1.10. We note that residual relatedness between the reduced set of sibships or between "unrelated" individuals is roughly comparable to first-degree and higher-order cousins, and this residual relatedness is reflected in the genomic control inflation factor λ=1.10. Since minimizing relatedness in the cohort also reduces inflation of the association test statistic, we conclude that excess relatedness is the major source of score inflation in our association study.
