# Supplementary material for: Genome-Wide Association Studies in an Isolated Founder Population from the Pacific Island of Kosrae
Source: PLoS Genet. 2009 Feb 6;5(2):e1000365. doi: 10.1371/journal.pgen.1000365 (PMC2628735; doi:10.1371/journal.pgen.1000365)
Supplement: Figure S3 — Quantile-quantile plots showing genome-wide association results for 15 quantitative traits. For each trait, the number of individuals used in the analysis, heritability, and genomic control correction factor (lambda) are given. (0.17 MB PDF) [file pgen.1000365.s003.pdf]

Symbol	Trait	N	h^2	λ
BMI	Body Mass Index	2,073	0.473	1.41
HT	Height	2,071	0.790	1.92
LEP	Leptin	2,435	0.196	1.31
pctfat	% body fat	1,446	0.414	1.32
WST	Waist Circumference	2,113	0.430	1.45
WT	Weight	2,046	0.520	1.42
DBP	Diastolic Blood Pressure	2,198	0.289	1.25
SBP	Systolic Blood Pressure	2,199	0.243	1.22
HDL	HDL-C and ApoA1	2,368	0.391	2.05
LDL	LDL-C and ApoB	2,367	0.414	1.64
TC	Total Cholesterol	2,346	0.425	1.49
TG	Triglycerides	2,348	0.274	1.20
FPG	Fasting plasma glucose	1,368	0.188	1.10
TSH	Thyroid Stimulating Hormone	1,502	0.272	1.47
CRP	C-reactive Protein	1,521	0.245	1.22

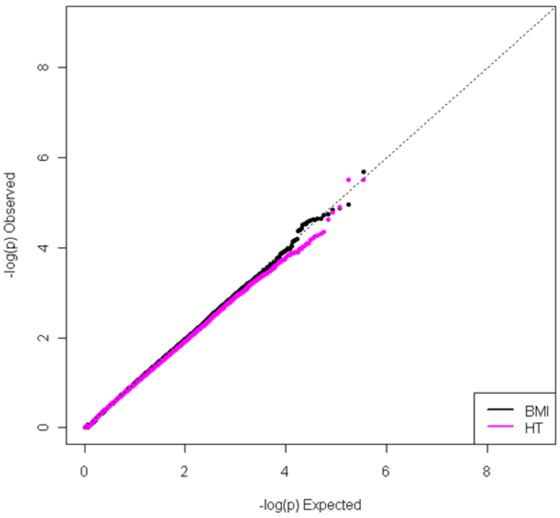

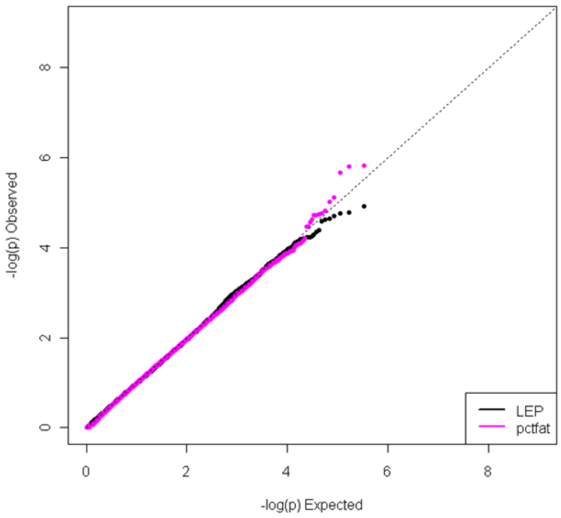

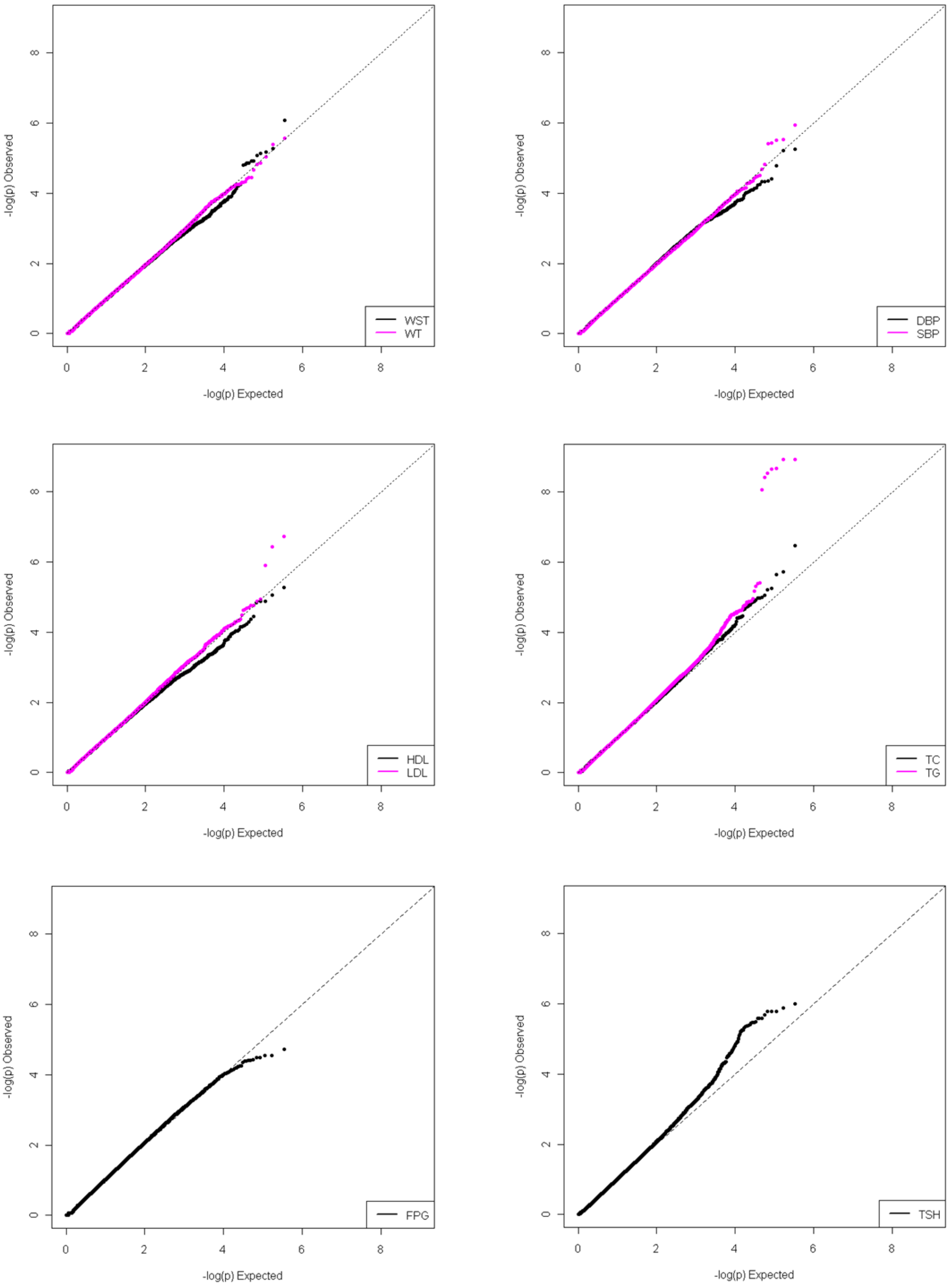
-log(p) Observed
-log(p) Expected
0
2
4
6
8
WST
WT
DBP
SBP
HDL
LDL
TC
TG
FPG
TSH

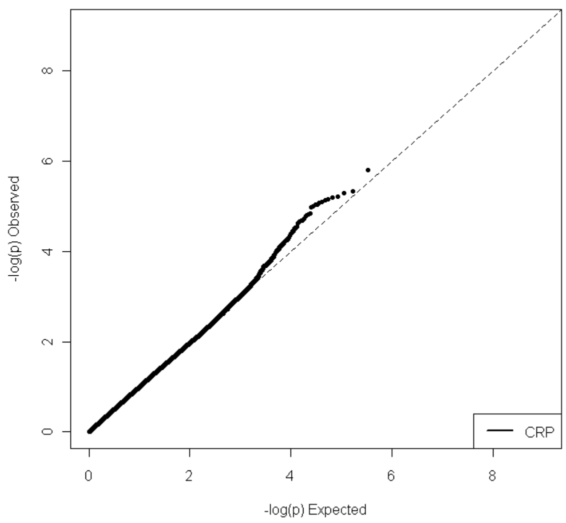

-log(p) Observed
-log(p) Expected
0
2
4
6
8
CRP
