# Supplementary material for: Genome-Wide Association Studies in an Isolated Founder Population from the Pacific Island of Kosrae
Source: PLoS Genet. 2009 Feb 6;5(2):e1000365. doi: 10.1371/journal.pgen.1000365 (PMC2628735; doi:10.1371/journal.pgen.1000365)
Supplement: Table S1 — Genotyping statistics for the Affymetrix 500 k assay. (0.04 MB DOC) [file pgen.1000365.s004.doc]

Table S1

| Assay | N | Mean call rate (%) | Median call rate (%) | Range (%) |
| --- | --- | --- | --- | --- |
| 500k Sty | 2,906 | 98.0 | 98.0 | 95.0-99.8 |
| 500k Nsp | 2,906 | 99.0 | 99.2 | 95.2-99.8 |
