# Supplementary material for: Genome-Wide Association Studies in an Isolated Founder Population from the Pacific Island of Kosrae
Source: PLoS Genet. 2009 Feb 6;5(2):e1000365. doi: 10.1371/journal.pgen.1000365 (PMC2628735; doi:10.1371/journal.pgen.1000365)
Supplement: Dataset S1 — Supplemental Trait Descriptions. (0.81 MB DOC) [file pgen.1000365.s005.doc]

**BMI**

Calculation: weight (kg) / height (m)^2^

Measurement:

Weight

1994 cohort, pounds

2001 cohort, kg converted to pounds

Height

1994 cohort, inches

2001 cohort, cm converted to inches

Trait distribution

Raw BMI (kg/m^2^)

| Sample* | N | Mean | SD | Median |
| --- | --- | --- | --- | --- |
| All | 2,073 | 31.29 | 5.67 | 30.92 |
| Males | 850 | 30.13 | 5.12 | 29.82 |
| Females | 1,223 | 32.09 | 5.89 | 31.68 |

* genotyped individuals included in the 500k analysis. Average BMI is used for individuals examined more than once.

**Males**

**Females**

Covariates: age, sex

Description of trait modeling:

Males age 24-80 and females age 22-80 were considered as having reached their full adult height and were included in the BMI analyses. BMI was log transformed to approximate a normal distribution. Data were separated into four groups based on screening year and gender. Following age correction, Z-scores were calculated for each group. For monozygotic twins and individuals screened twice, the average Z-score was used. Z-scores were pooled across groups and used for association analyses.

Additional comments

Lambda 1.41

Heritability 0.473

**Height (HT)**

Calculation: n/a

Measurement:

Height

1994 cohort, inches

2001/3 cohort, cm converted to inches

Trait distribution

Raw Height (inches)

| Sample* | N | Mean | SD | Median |
| --- | --- | --- | --- | --- |
| All | 2,071 | 62.17 | 3.27 | 61.87 |
| Males | 846 | 65.08 | 2.25 | 65.00 |
| Females | 1,225 | 60.16 | 2.17 | 60.25 |

* genotyped individuals included in the 500k analysis. Average height is used for patients examined more than once.

**Males**

**Females**

Covariates: age, sex

Description of trait modeling:

Males age 24-80 and females age 22-80 were considered as having reached their full adult height. Individuals measured more than once were omitted from the analysis if their measurements differed by more than two inches. Height was log transformed to approximate a normal distribution. Males and females were considered separately. Following age correction, Z-scores were calculated for each gender. For monozygotic twins and patients screened more than once, the average Z-score was used. Z-scores were pooled for males and females and used for association analyses.

Additional comments

Lambda 1.85

Heritability 0.790

**Leptin (LEP)**

Calculation: n/a

Measurement:

1994: Measured in the Friedman lab, The Rockefeller University, by ELISA (Diagnostic Systems Laboratories, Webster, TX)

2001/2003: Measured in the Friedman lab, The Rockefeller University, by ELISA (Diagnostic Systems Laboratories, Inc, Webster, TX, and Linco Research Inc, St. Charles, MO)

Trait distribution


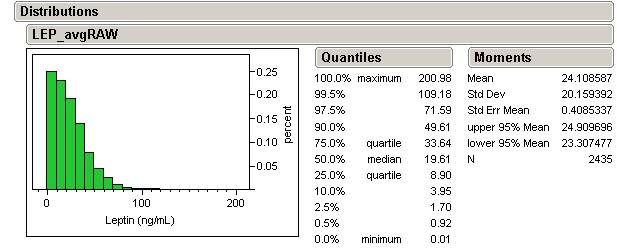


Raw Leptin (ng/mL)

| Sample* | N | Mean | SD | Median |
| --- | --- | --- | --- | --- |
| All | 2,435 | 24.11 | 20.16 | 19.61 |
| Males | 1,017 | 11.46 | 11.01 | 8.63 |
| Females | 1,418 | 31.18 | 20.34 | 29.44 |

* genotyped individuals included in the 500k analysis. Average leptin is used for individuals examined more than once.

**Males**


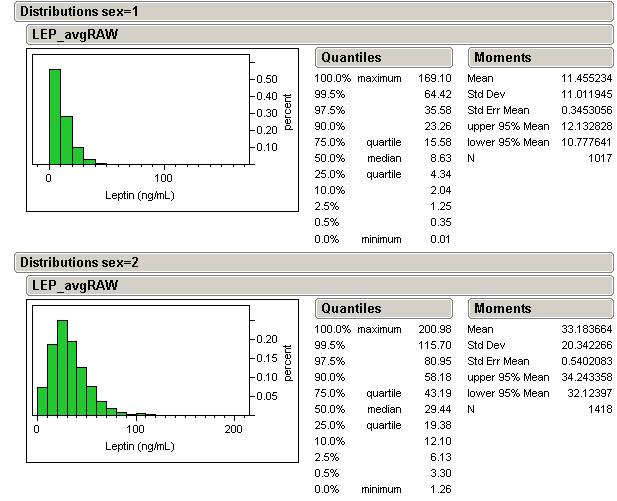


**Females**


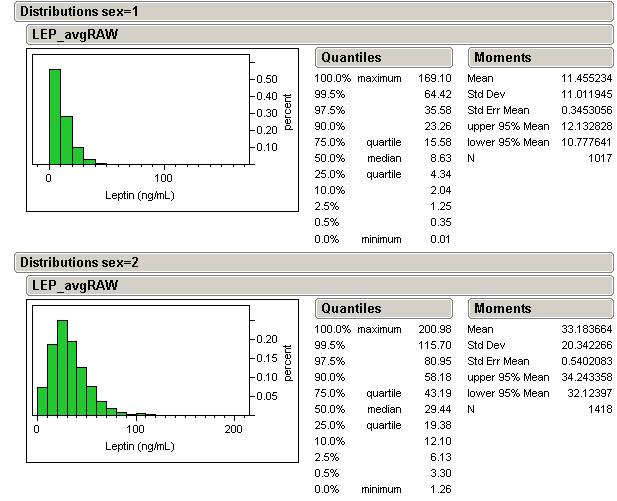


Covariates: age, sex, BMI

Description of trait modeling:

Leptin was square-root transformed to approximate a normal distribution. Data were separated into four groups based on screening year and gender. Following correction for age and BMI, Z-scores were calculated for each group. For monozygotic twins and individuals screened more than once, the average Z-score was used. Z-scores were pooled across groups and used for association analyses.

Additional comments

Lambda 1.31

Heritability 0.196

**Percent body fat (PCTFAT)**

Calculation: n/a

Measurement: custom equipment, Tanita manufacturing company, Tokyo

Trait distribution

Total body fat (%)

| Sample* | N | Mean | SD | Median |
| --- | --- | --- | --- | --- |
| All | 1,446 | 30.37 | 10.26 | 31.72 |
| Males | 542 | 21.055 | 7.95 | 22.39 |
| Females | 904 | 35.95 | 6.88 | 36.85 |

* genotyped individuals included in the 500k analysis.

**Males**

**Females**

Covariates: age, sex

Description of trait modeling:

%fat was log transformed to approximate a normal distribution. Data were separated by gender. Following age correction, Z-scores were calculated for each gender. For monozygotic twins, the average Z-score was used. Z-scores were pooled for males and females and used for association analyses.

Additional comments

Lambda 1.32

Heritability 0.414

**Waist circumference (WST)**

Calculation: n/a

Measurement:

1994, inches

2001, cm converted to inches

Trait distribution

Trait distribution

Waist Circumference (inches)

| Sample* | N | Mean | SD | Median |
| --- | --- | --- | --- | --- |
| All | 2,113 | 37.49 | 5.01 | 37.00 |
| Males | 858 | 37.19 | 4.66 | 37.00 |
| Females | 1,255 | 37.71 | 5.22 | 37.50 |

* genotyped individuals included in the 500k analysis.

**Males**

**Females**

Covariates: age, sex

Description of trait modeling:

Males age 24-80 and females age 22-80 were considered as having reached their full adult growth. Waist circumference was log transformed to approximate a normal distribution. Males and females were considered separately. Following age correction, Z-scores were calculated for each gender. For monozygotic twins and patients screened more than once, the average Z-score was used. Z-scores were pooled for males and females and used for association analyses.

Additional information

Lambda 1.45

Heritability 0.430

**Weight (WT)**

Calculation: n/a

Measurement:

Weight

1994 cohort, pounds

2001 cohort, kg converted to pounds

Trait distribution

Raw Weight (lb)

| Sample* | N | Mean | SD | Median |
| --- | --- | --- | --- | --- |
| All | 2,045 | 172.28 | 34.70 | 169.6 |
| Males | 849 | 181.90 | 34.70 | 179.2 |
| Females | 1,196 | 165.46 | 33.05 | 163.1 |

* genotyped individuals included in the 500k analysis. Average weight is used for individuals screened in 1994 and 2001.

**Males**

**Females**

Covariates: age, sex

Description of trait modeling:

Males age 24-80 and females age 22-80 were included in the analysis. Weight was log transformed to approximate a normal distribution. Data were separated into four groups based on screening year and gender. Following age correction, Z-scores were calculated for each group. For monozygotic twins and individuals screened twice, the average Z-score was used. Z-scores were pooled across groups and used for association analyses.

Additional comments

Lambda 1.42

Heritability 0.520

**High Density Lipoprotein Cholesterol (HDL–C) and Apolipoprotein A1 (ApoA1)**

**Measurement:**

ApoA1: Measured using a standard double antibody immunoassay at the Youngman Laboratory at Oxford, England.

HDL-C : measured on a Roche COBAS Integra clinical chemistry analyzer (Roche Diagnostics, Indianapolis, IN) at The Rogosin Institute (New York, NY) using a direct method utilizing PEG-modified enzymes that only react with HDL-C.

**Trait distribution**

Raw ApoA1 levels – 1994 (mg/dL)

| Sample* | N | Mean | SD | Median |
| --- | --- | --- | --- | --- |
| All | 1,662 | 117.0 | 24.2 | 115.4 |
| Males | 699 | 108.1 | 21.9 | 106.4 |
| Females | 963 | 123.5 | 23.7 | 121.3 |

* genotyped individuals included in the 500k analysis.

Male

Female

| Sample* | N | Mean | SD | Median |
| --- | --- | --- | --- | --- |
| All | 1,576 | 38.3 | 10.7 | 37 |
| Males | 593 | 33.4 | 9.2 | 32 |
| Females | 983 | 41.3 | 10.5 | 40 |

Raw HDL-C levels – 2001 (mg/dL)

*genotyped individuals included in the 500k analysis.

Male

Female

Description of trait preparation for analysis:

Both ApoA1/1994 and HDL-C/2001 were each separated by gender and log transformed to approximate a normal distribution. Individuals more than 3 standard deviations from the mean were excluded. Following age correction, Z-scores were calculated for each group. For monozygotic twins, the average Z-score was used. For 870 individuals who were screened in both surveys, ApoA1/1994 (117.31 mg/dL ± 24.44) and HDL-C/2001 (38.96 mg/dL ± 11.03) Z-scores were averaged. Z-scores were pooled across groups and used for association analyses.

Additional comments

Lambda: 2.05

Heritability 0.391

**Low Density Lipoprotein Cholesterol (LDL-C) and** **Apolipoprotein B (ApoB)**

**Measurement:**

Apo B was measured in the 1994 cohort by a double-antibody immunoassay technique at the Youngman Laboratory at Oxford University.

LDL-C was calculated in the 2001 cohort by using the Friedewald formula. For individuals with a triglyceride value >400 mg/dL the LDL-C value was set as missing.

**Trait distribution ApoB**

Raw ApoB (mg/dl)

| Sample* | N | Mean | SD | Median |
| --- | --- | --- | --- | --- |
| All | 1,661 | 87.1 | 21.0 | 86.5 |
| Males | 700 | 89.5 | 21.3 | 89.4 |
| Females | 961 | 85.3 | 20.6 | 84.2 |

* genotyped individuals included in the 500k analysis.

Males Females


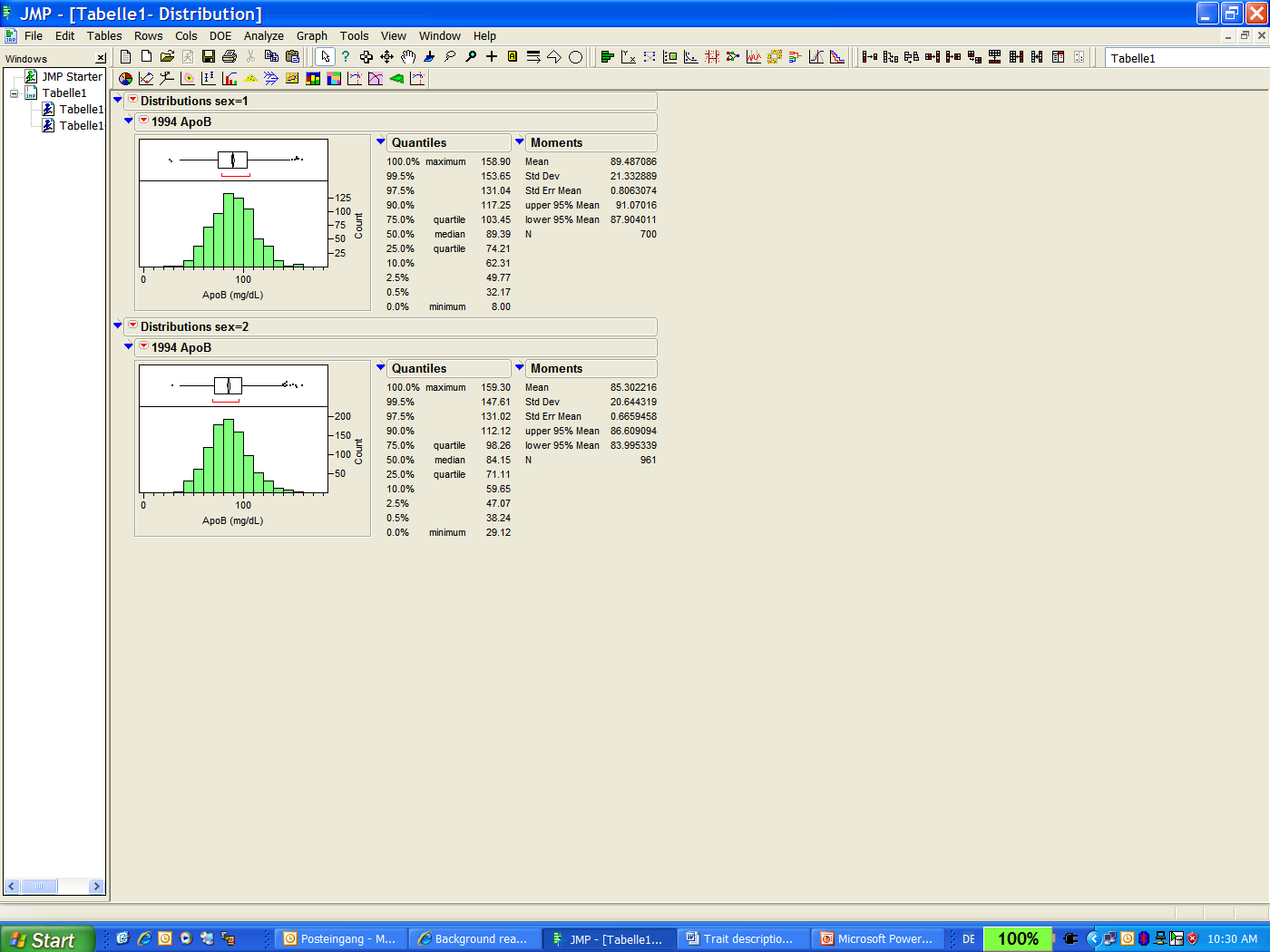

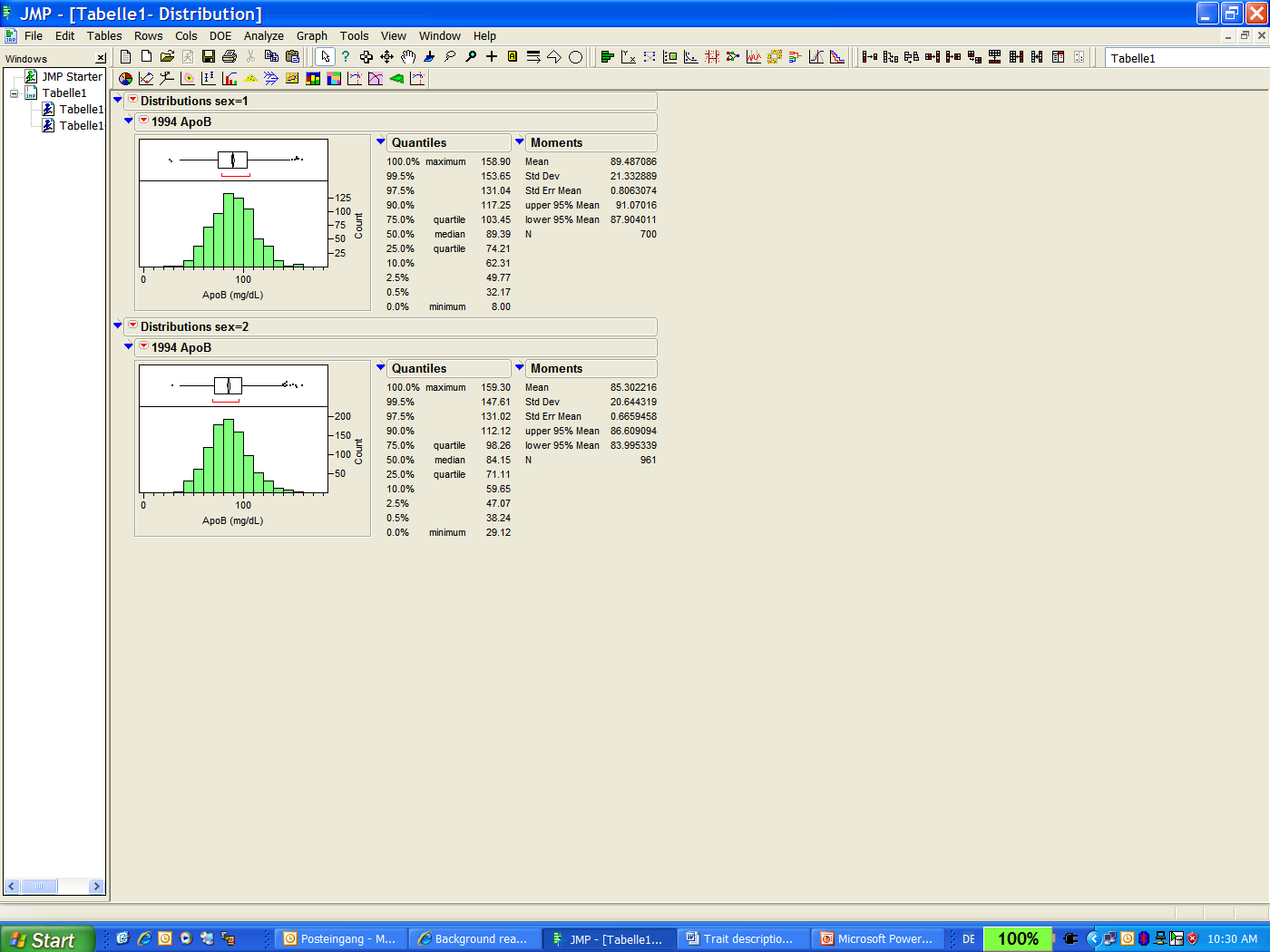


**Trait distribution LDL-C**

Raw LDL-C (mg/dl)

| Sample* | N | Mean | SD | Median |
| --- | --- | --- | --- | --- |
| All | 1,579 | 102.8 | 28.8 | 100.0 |
| Males | 598 | 103.8 | 31.2 | 101.0 |
| Females | 981 | 102.2 | 27.3 | 100.0 |

* genotyped individuals included in the 500k analysis.

Males Females


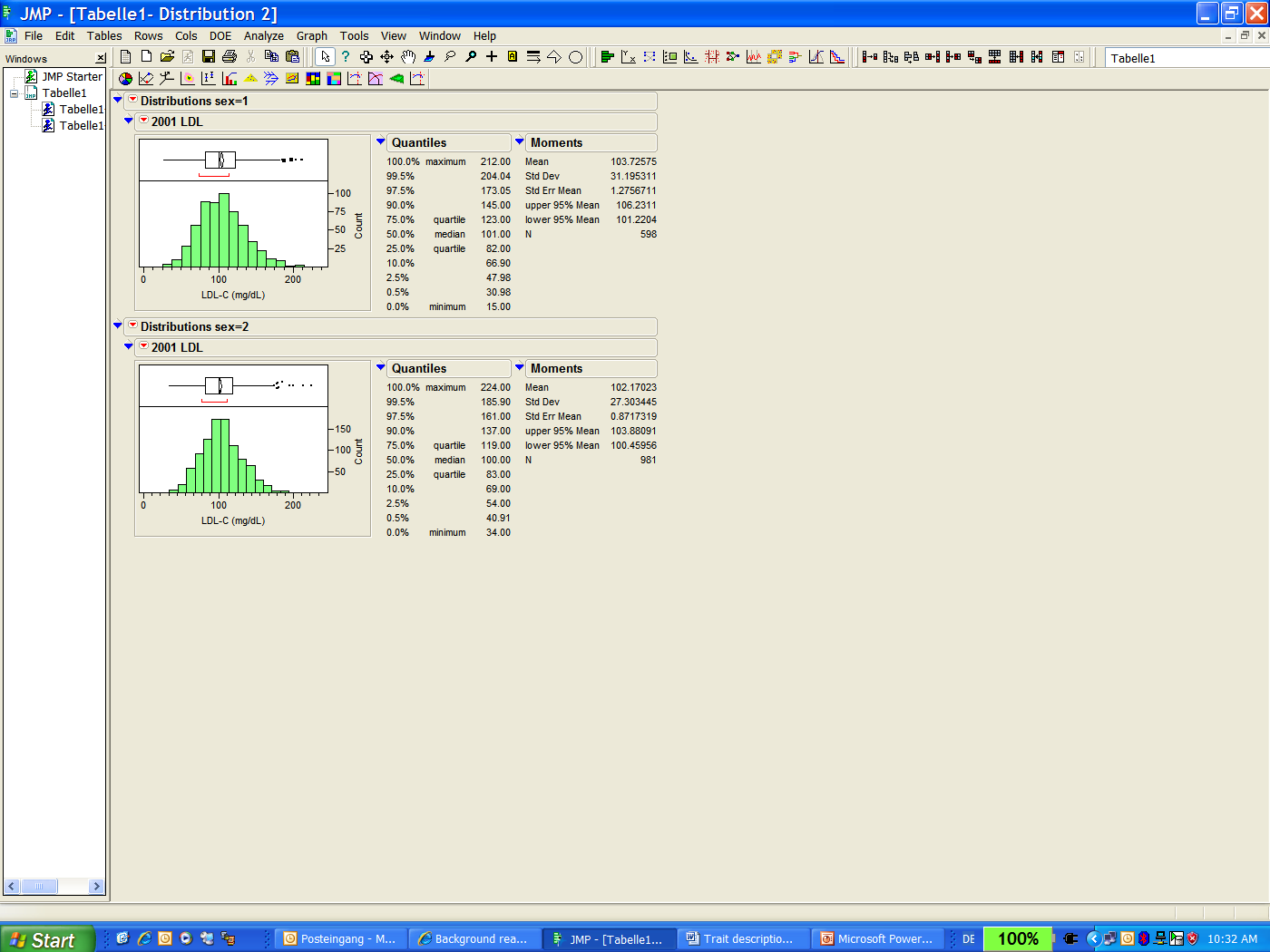

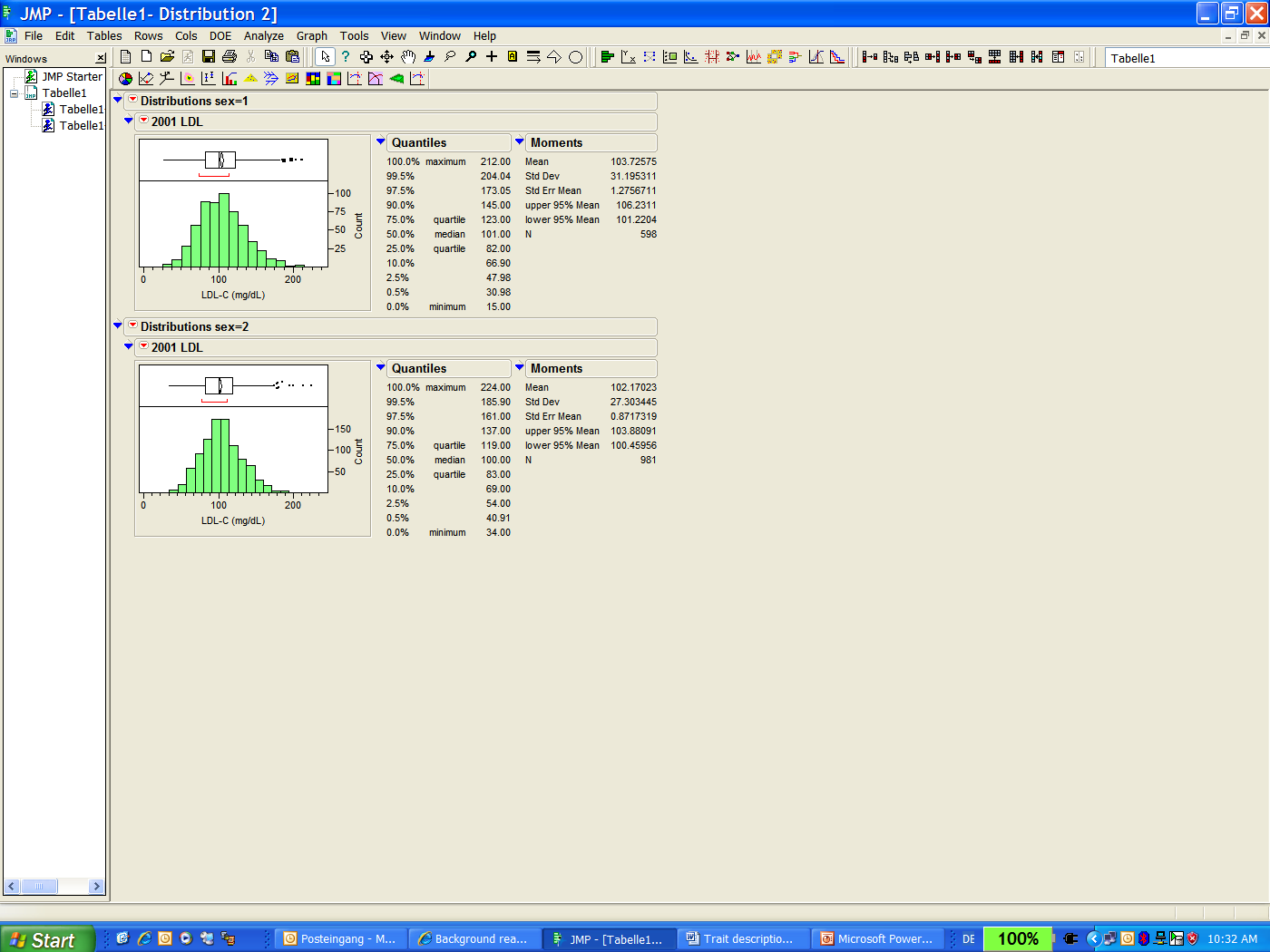


**Description of trait modeling:**

Both ApoB/1994 and LDL-C/2001 were each separated by gender and square root transformed to approximate a normal distribution. Individuals more than 3 standard deviations from the mean were excluded. Following age correction, Z-scores were calculated for each group. For monozygotic twins, the average Z-score was used. For 875 individuals who were screened in both surveys, ApoB/1994 (mean: 85.95 mg/dL ± 21.09) and LDL-C/2001 (mean: 111.04 mg/dL ± 27.95) Z-scores were averaged. Z-scores were pooled across groups and used for association analyses.

Additional comments

Lambda: 1.64

Heritability 0.414

**Total Cholesterol (TC)**

Measurement: mg/dL

1994: measured in the Breslow lab, The Rockefeller University, using an enzymatic colorimetric assay (Boehringer-Mannheim, Germany)

2001: measured at The Rogosin Institute (New York, NY) on a Roche COBAS Integra clinical chemistry analyzer using an enzymatic method

Trait distribution of raw TC values (mg/dL):

| Sample* | N | Mean | SD | Median |
| --- | --- | --- | --- | --- |
| All | 2,346 | 167.1 | 32.8 | 166.0 |
| Males | 972 | 166.9 | 34.7 | 166.0 |
| Females | 1,374 | 167.3 | 31.5 | 165.0 |

*All individuals genotyped in the 500K analysis. For individuals screened more than once, averaged raw TC values are used.

**Males Females**


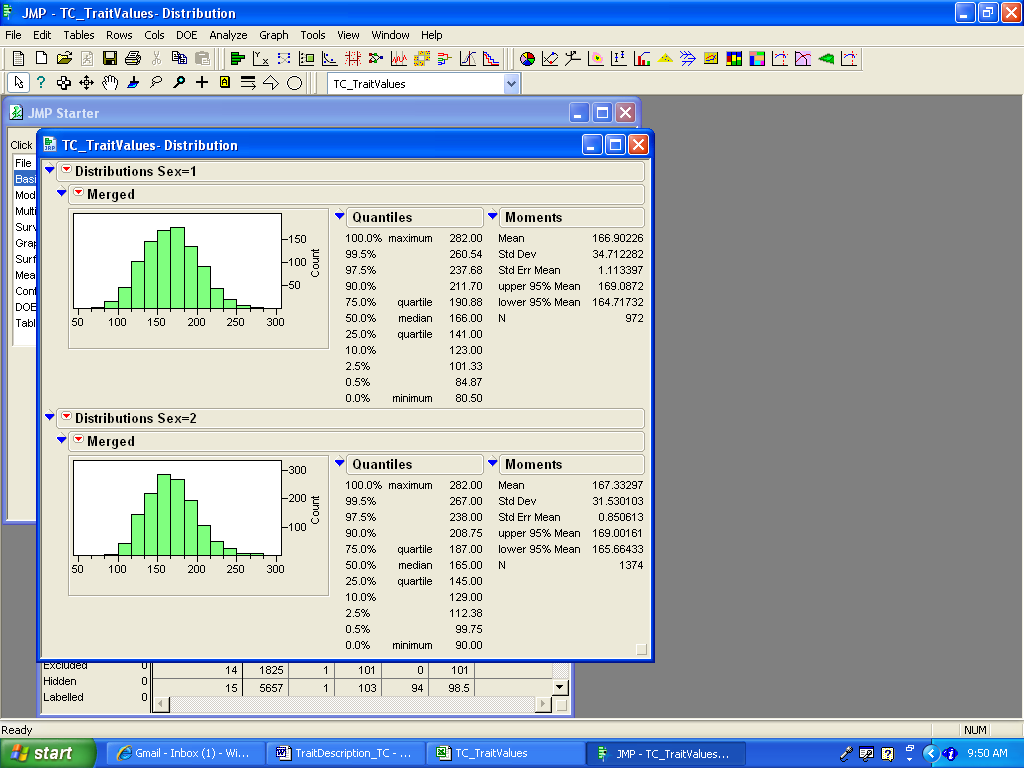

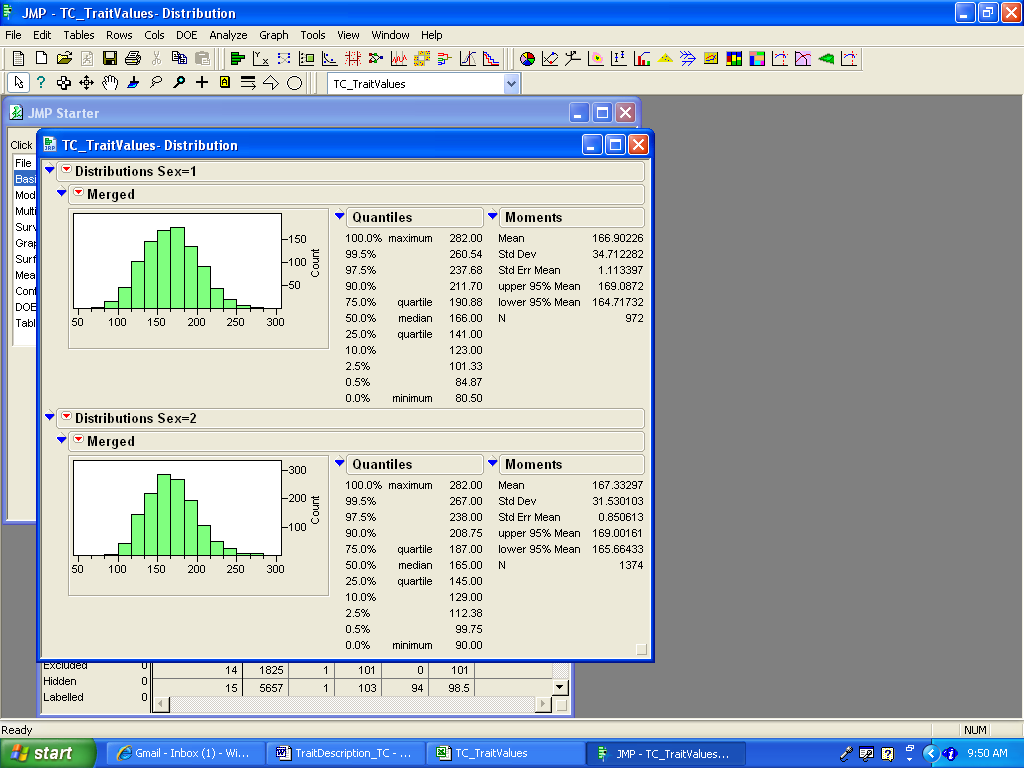


Total Cholesterol (mg/dL)

Total Cholesterol (mg/dL)

Description of trait preparation for analysis:

The sample was separated into four groups by sex and by screening year. Within each group, total cholesterol values were natural log transformed to approximate a normal distribution and individuals more than 3 standard deviations from the mean were excluded. Total cholesterol values were corrected for age and z-scores were calculated for each group. Z-scores were averaged for monozygotic twins and for individuals screened in both 1994 and 2001. Z-scores from the four groups were pooled and used for association analysis.

Additional comments:

Lambda: 1.49

Heritability 0.425

**Triglycerides (TG)**

Measurement: mg/dL

1994: measured in the Breslow lab, The Rockefeller University, using an enzymatic colorimetric assay (Sigma-Aldrich)

2001: measured at the Rogosin Institute on a Roche COBAS Integra clinical chemistry analyzer, using a standard enzymatic method

Trait distribution of raw TG values (mg/dL):

| Sample* | N | Mean | SD | Median |
| --- | --- | --- | --- | --- |
| All | 2,348 | 100.1 | 44.3 | 88.5 |
| Males | 977 | 111.5 | 52.5 | 98.0 |
| Females | 1,371 | 92.0 | 35.3 | 83.5 |

*All individuals genotyped in the 500K analysis. For individuals screened more than once, averaged raw TG values are used.

**Males Females**


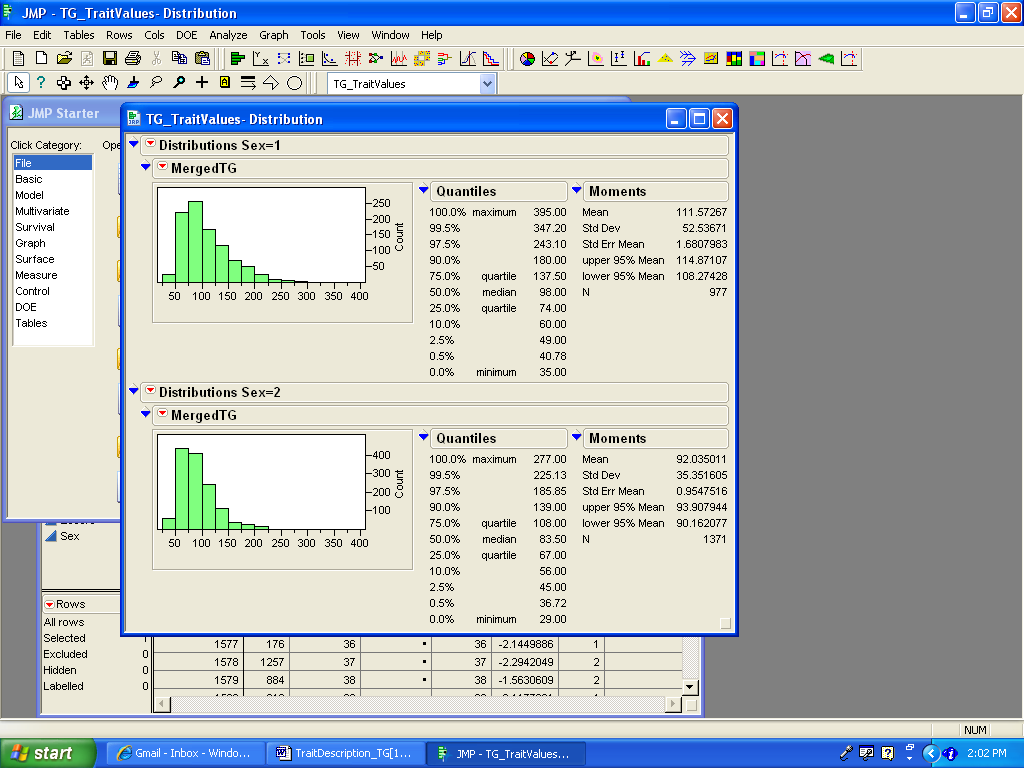

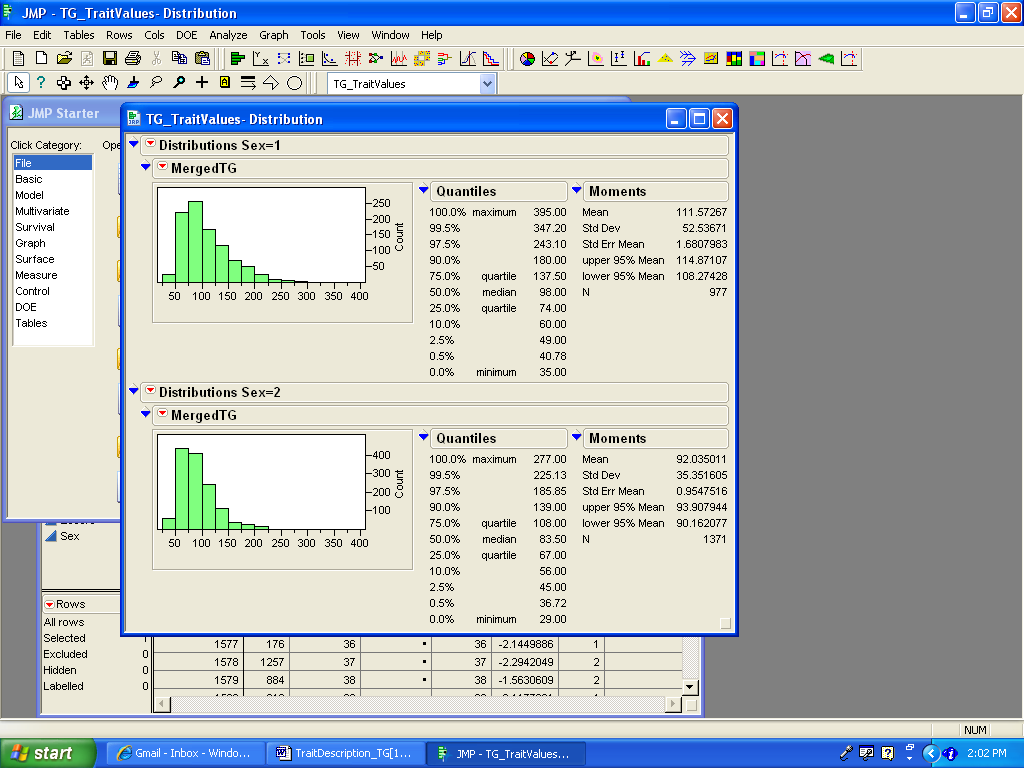


Triglycerides (mg/dL)

Triglycerides (mg/dL)

Description of trait preparation for analysis:

The sample was separated into four groups by sex and by screening year. Within each group, triglycerides were log transformed to approximate a normal distribution and individuals more than 3 standard deviations from the mean were excluded. Triglycerides were corrected for age and z-scores were calculated for each group. Z-scores were averaged for monozygotic twins and for individuals screened in both 1994 and 2001. Z-scores from the four groups were pooled and used for association analysis.

Additional comments:

Lambda: 1.20

Heritability 0.274

**Systolic blood pressure (SBP)**

Calculation: n/a

Measurement:

SBP 1994 cohort, average of 3 seated blood measurements (mmHg)

2001 cohort, average of 2 seated blood measurements (mmHg)

Trait distribution

Raw SBP (mmHg)

| Sample* | N | Mean | SD | Median |
| --- | --- | --- | --- | --- |
| All | 2,198 | 117.36 | 17.11 | 115.0 |
| Males | 908 | 120.42 | 15.71 | 120.0 |
| Females | 1,290 | 115.20 | 17.73 | 111.5 |

* genotyped individuals included in the 500k analysis. Average SBP is used for individuals examined more than once.

**Males**

**Female**

Description of trait modeling:

Males and females age 20-75 were included in the blood pressure analyses (mean age = 41.5, male; 41.0, female). Data were separated into four groups based on screening year and gender. Treatment for hypertension was taken into account by calculating an adjusted residual based on **Levy et. al. Hypertension 36: 477-483, 2000**. Following age and BMI adjustment by linear regression which was performed separately from unique subjects of 1994 or 2001 cohorts, the standardized residuals were used as quantitative traits. The standard residuals for individuals examined more than once (both 1994 and 2001 screens available) were obtained using the average age and BMI for adjustment. For monozygotic twins and individuals screened twice, the average standardized residuals were used. The standardized residuals were pooled across groups and used for association analyses.

Additional comments

Lambda 1.22

Heritability 0.243

**Diastolic blood pressure (DBP)**

Calculation: n/a

Measurement:

DBP 1994 cohort, average of 3 seated blood measurements (mmHg)

2001 cohort, average of 2 seated blood measurements (mmHg)

Trait distribution

Raw Weight (mmHg)

| Sample* | N | Mean | SD | Median |
| --- | --- | --- | --- | --- |
| All | 2,198 | 77.25 | 10.45 | 77.5 |
| Males | 908 | 79.62 | 10.17 | 80.0 |
| Females | 1,290 | 75.59 | 10.33 | 75.0 |

* genotyped individuals included in the 500k analysis. Average DBP is used for individuals screened in 1994 and 2001.

**Males Female**

Description of trait modeling:

Males and females age 20-75 were included in the blood pressure analyses (mean age = 41.5, male; 41.0, female). Data were separated into four groups based on screening year and gender. Treatment for hypertension was taken into account by calculating an adjusted residual based on **Levy et. al. Hypertension 36: 477-483, 2000**. Following age and BMI adjustment by linear regression, which was performed separately from unique subjects of the 1994 or 2001 cohorts, the standardized residuals were used as quantitative traits. The standard residuals for individuals examined more than once (both 1994 and 2001 screens available) were obtained using the average age and BMI for adjustment. For monozygotic twins and individuals screened twice, the average standardized residuals were used. The standardized residuals were pooled across groups and used for association analyses.

Additional comments

Lambda 1.25

Heritability 0.289

**Fasting plasma glucose (FPG)**

Measurement: Fasting plasma glucose (mg/dL)

1994, Glucometer Elite (Bayer Healthcare, Tarrytown, NY)

2001, venous blood (The Rogosin Institute, New York, NY)

Trait distribution

Fasting plasma glucose (mg/dL)

| Sample* | N | Mean | SD | Median |
| --- | --- | --- | --- | --- |
| All | 1,368 | 85.01 | 8.07 | 86.00 |
| Males | 617 | 85.46 | 8.37 | 86.00 |
| Females | 751 | 84.65 | 7.81 | 85.00 |

* genotyped individuals included in the 500k analysis.

**Males**

**Females**

Covariates: age, sex, BMI

Description of trait modeling

Euglycemic individuals (FBG < 100 mg/dL) with no history of diabetes were included in the association analysis for Fasting plasma glucose. Fasting plasma glucose was measured on the island by hand-held glucometer in 1994 and 2001. For patients screened more than once, only the most recent clinical data were used. In the 2001 screening, Fasting plasma glucose was also measured from venous blood. Measurements from venous blood were used whenever possible, although subjects were excluded if the glucometer and venous blood measurements differed by 20%.

Log-transformed FBG was separated by gender and adjusted for age and BMI. Z-scores were calculated separately for each gender, then pooled for the association analysis.

Additional comments

Lambda 1.10

Heritability 0.188

**Thyroid Stimulating Hormone (TSH)**

Calculation: n/a

Measurement:

μIU/ml, by The Rogosin Institute, New York, NY

Trait distribution

Thyroid Stimulating Hormone (mIU/L)

| Sample* | N | Mean | SD | Median |
| --- | --- | --- | --- | --- |
| All | 1,502 | 1.59 | 0.90 | 1.39 |
| Males | 581 | 1.57 | 0.86 | 1.39 |
| Females | 921 | 1.61 | 0.93 | 1.40 |

* genotyped individuals included in the 500k analysis.

**Males**

**Females**

Covariates: sex

Description of trait modeling:

TSH was measured for the 2001/2003 cohort only. No significant effect of age or BMI was observed on raw TSH values. TSH was log transformed to approximate a normal distribution. Z-scores were calculated for each gender separately and genders were pooled prior to association analysis.

Additional comments:

Lambda 1.47

Heritability 0.272

**C-Reactive Protein (CRP)**

Calculation: n/a

Measurement:

mg/L, measured using a chemiluminescent immunometric assay (Immulite 2000 High Sensitivity CRP, DPC, Los Angeles, CA) by The Rogosin Institute, New York, NY.

Trait distribution

C-Reactive Protein (mg/L)

| Sample* | N | Mean | SD | Median |
| --- | --- | --- | --- | --- |
| All | 1,521 | 2.95 | 3.53 | 1.70 |
| Males | 572 | 2.78 | 3.53 | 1.50 |
| Females | 949 | 3.04 | 3.52 | 1.80 |

* genotyped individuals included in the 500k analysis.

**Males**

**Females**

Covariates: age, BMI

Description of trait modeling:

Positive outliers (>3 SD corresponding to >27.4 mg/L) were considered acute inflammatory reactions and excluded (n=28). All values below the functional sensitivity level of the assay (0.02) were Winsorized to 0.02 (n=36). Values were log transformed. CRP was adjusted for age and BMI using linear regression. No significant effect of sex was observed (P>0.05).

Additional comments:

Lambda 1.22

Heritability 0.245
